# Elucidating the Degradation Behavior of a 25 cm^2^ Pure‐Water‐Fed Non‐Precious Metal Anion Exchange Membrane Water Electrolyzer Cell

**DOI:** 10.1002/smll.202506262

**Published:** 2025-12-03

**Authors:** Michelle S. Lemcke, Robert Göckeritz, Alexander Müller, Kerstin Witte‐Bodnar, Wolfram Münchgesang, Michael Bron

**Affiliations:** ^1^ Electrochemical Analytics Fraunhofer Institute for Wind Energy Systems IWES 27572 Bremerhaven Germany; ^2^ Institute of Chemistry Martin Luther University Halle‐Wittenberg 06120 Halle (Saale) Germany; ^3^ Material Diagnostics for H2 Technologies Fraunhofer Institute for Microstructure of Materials and Systems IMWS 06120 Halle (Saale) Germany; ^4^ Applied Biosciences and Process Engineering, Hochschule Anhalt University of Applied Science 06366 Köthen Germany

**Keywords:** anion exchange membrane water electrolysis, degradation, earth‐abundant transition metal, electrochemical impedance spectroscopy, electrolyzer durability

## Abstract

Anion exchange membrane water electrolysis (AEMWE) has become a promising technology for generating hydrogen in a carbon‐neutral economy. However, its competitiveness is currently limited by the low durability of AEMWE systems. To increase its durability and to advance its industrial application, this study examines the degradation behavior of a 25 cm^2^ AEMWE cell utilizing non‐precious metal catalysts and pure water feed in a short‐term durability test. Polarization curves and electrochemical impedance spectroscopy, along with scanning electron microscopy and energy‐dispersive X‐ray spectroscopy, are employed to identify factors affecting cell efficiency and stability. The pure‐water‐fed AEMWE cell exhibits high performance losses and low stability that is mainly related to degradation in the anode and at the interface of anode and membrane. Ionomer degradation, reducing hydroxide ion conductivity, and mechanical stability of the catalyst layer, is identified as the key factor decreasing cell efficiency and stability during pure water operation. The findings aim to guide the development of strategies to enhance cell performance and durability of pure‐water‐fed AEMWE.

## Graphical abstract

The degradation behavior of a pure‐water‐fed non‐precious metal 25 cm^2^ AEMWE cell is investigated in a short‐term durability test. Factors affecting cell efficiency and stability are identified by electrochemical characterization accompanied by morphological and elemental studies. This contributes to the development of a more stable and efficient AEMWE.

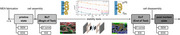

## 1 Introduction

Hydrogen production through water splitting powered by renewable energies is key to realizing a carbon‐neutral economy. Among the various electrolyzer technologies, anion exchange membrane water electrolysis (AEMWE) has attracted considerable attention due to its potential for cost‐effective and efficient hydrogen production. Combining the advantages of alkaline electrolysis (AEL) and proton exchange membrane water electrolysis (PEMWE), AEMWE is featured by a compact zero‐gap cell design utilizing an anion‐selective polymer membrane, non‐precious metal catalysts and other inexpensive materials for electrolyzer components due to the alkaline environment. This reduces costs compared to PEMWE while enabling advantages of PEMWE such as high current densities and variable‐load operation which is beneficial when powered by wind and solar energy.^[^ ^1^, ^2^, ^3^, ^4^, ^5^, ^6^, ^7^, ^8^ ^]^

Nevertheless, further research and development is still required to advance the AEMWE technology. A major limiting factor for the scale‐up and commercialization of AEMWE is the durability of the system.^[^ ^6^, ^9^, ^10^ ^]^ Recent proposals of the Clean Hydrogen Partnership aim to reduce electricity consumption and capital cost, while increasing the nominal current density and lifetime (see **Table** **1**).^[^ ^11^ ^]^To enhance cell efficiency and durability, a significant amount of effort has been dedicated to developing catalysts and anion exchange membranes (AEMs). A few achievements are highlighted in Table 1. However, most studies use a supporting liquid electrolyte such as a dilute KOH feed to compensate the significant performance and stability issues caused by instable AEMs and ionomers in pure‐water‐fed AEMWE systems.^[^ ^10^, ^17^, ^18^ ^]^ Although the supporting electrolyte enhances reaction kinetics and ionic conductivity compared to a pure water feed, it also introduces limitations found in AEL like shunt currents and higher balance‐of‐plant costs.^[^ ^10^, ^19^ ^]^

**Table 1:** AEMWE targets for 2030 and studies on AEMWE showing the current status regarding cell efficiency and durability.

	j	Operation time	Degradation rate	Degradation rate	Cell size	T	Supporting
Study	/A cm^−2^	/h	/$\mu Vh^{-1}$	/% per 1000 h	/cm^2^	/°C	electrolyte
Target 2030 [11]	1.5			0.5			
1 [12]	1.0	10000	1	0.2	5	60	1.0 M KOH
2a [13]	0.2	8900	18	0.9	50	70	1.0 M KOH
2b [13]	0.6	5000	13	0.7	50	70	1.0 M KOH
3 [14]	0.5	1000	200	13	6.25	60	1.0 M KOH
4 [15]	1.0	600	100	6	5	60	Pure water
5 [16]	1.0	550	71	4	4	80	Pure water

Only a few reports on AEMWE address the operation with solely pure water. The majority exhibits low current densities and short operating times.^[^ ^4^, ^5^, ^6^, ^20^ ^]^ Recent research provides improved cell performance in pure water, achieving 1 A cm^−2^ below a cell voltage of 2.0 V.^[^ ^14^, ^17^, ^21^, ^22^, ^23^, ^24^ ^]^ However, these AEMWE systems typically contain precious metal catalysts and are featured by a small cell size with electrode areas of 1 to 5 cm^2^. Additionally, some did not perform stability tests at all^[^ ^14^, ^22^ ^]^ and others conducted stability tests at mild conditions like a low current density of 0.2 A cm^−2^.^[^ ^21^, ^23^, ^24^ ^]^ These features and conditions are not application oriented. So far, only two pure‐water‐fed non‐precious metal AEMWE systems have been reported to maintain for around 600 h at 1 A cm^−2^ (see Table 1, Study 4 and 5).^[^ ^15^, ^16^ ^]^ Although these are promising results, they are below the 2030 targets from the Clean Hydrogen Partnership and there is still a long way to go to achieve competitive lifetimes of 10000 to 100000 h for large AEMWE systems.

To progress in industrially relevant AEMWE, it is essential to analyze and comprehend the performance and degradation behavior of AEMWE systems. In the present study, we, therefore, investigate AEMWE systems in a short‐term durablity test under application‐oriented conditions to reveal factors limiting high cell efficiency and durability. Special features of our research are the use of non‐precious metal membrane electrode assemblies (MEAs), larger electrode areas of 25 cm^2^ and a pure water feed. Polarization curves and electrochemical impedance spectroscopy (EIS) are carried out to analyze and evaluate the performance and degradation behavior of these AEMWE cells. Scanning electron microscopy (SEM) and energy‐dispersive X‐ray spectroscopy (EDS) are used to identify changes in morphology and material composition. In combination, the electrochemical and morphological analyses indicate causes for degradation and stability issues and relate them to cell components. This aims to guide the development of approaches to improve cell efficiency and durability to advance AEMWE.

## 2 Results and Discussion

**Figure 1:**
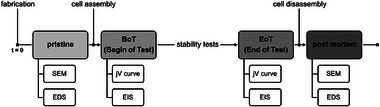
States of the membrane electrode assembly (MEA) and chronological sequence of the measurements including scanning electron microscopy (SEM), energy‐dispersive X‐ray spectroscopy (EDS), polarization curve (jV curve), electrochemical impedance spectroscopy (EIS) and stability tests.

**Figure** **1** illustrates the chronological sequence of the measurements and the states of the membrane electrode assembly (MEA). The MEA was developed for pure water operation and utilized a new membrane developed by Fumatech BWT GmbH placed between two non‐precious metal electrodes with an electrode area of 25 cm^2^. As anode electrode, copper‐cobalt‐manganese‐oxide was deposited on a modified 316L‐fiber felt, while the cathode electrode was made of Raney‐Nickel on carbon paper. Further information are given in the Experimental Section 4.

**Figure 2:**
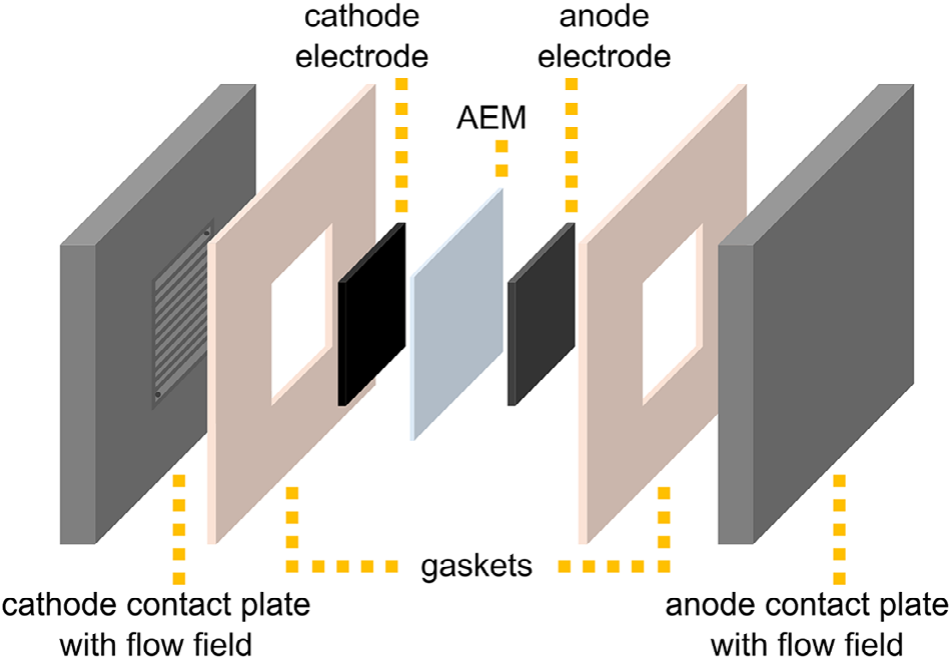
Schematic representation of the AEMWE single‐cell setup with an electrode area of 25 cm^2^ used in this study.

The state of the MEA after fabrication is referred to as the pristine state and was examined using scanning electron microscopy (SEM) and energy‐dispersive X‐ray spectroscopy (EDS) on one MEA sample. After fabrication, the MEA was installed in the AEMWE cell, schematized in **Figure** **2**, to perform the electrolysis test. Begin of test (BoT) represents the state of the MEA at the start of the electrolysis. It corresponds to the first electrochemical characterization of the AEMWE cell to determine the initial performance behavior of the AEMWE cell. The electrochemical characterization involved carrying out polarization curves and electrochemical impedance spectroscopy (EIS). Subsequently, stability tests were conducted to stress the AEMWE cell and to induce its degradation. This was followed by the final electrochemical characterization to determine the performance behavior at the end of test (EoT). Finally, after the MEA was disassembled from the cell, the MEA was analyzed by SEM and EDS to examine its post mortem state.

It is noted that BoT was presented and discussed in more detail in our previous work.^[^ ^25^ ^]^ The present study focuses on the degradation behavior of the AEMWE cell. Therefore, we start with the electrochemical evaluation highlighting changes between BoT and EoT. This is then complemented by morphological and elemental studies analyzing the pristine and post mortem state of the MEA for a deeper understanding of the occurred changes. Next, approaches to improve the AEMWE cell efficiency and stability are suggested. Finally, the performance and stability of the developed AEMWE cell is compared to the performance data of a commercially available one and to literature values.

### 2.1 Electrochemical Evaluation of AEMWE Cells

**Figure 3:**
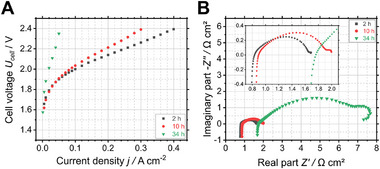
A) Polarization curves of the developed non‐precious metal AEMWE cell with an electrode area of 25 cm^2^ operated in pure water; B) Nyquist plots of the same AEMWE cell obtained from EIS at 2.0 V from 100 kHz to 100 mHz.

For a deeper insight into the electrochemical behavior, electrolysis tests were carried out in three distinct environments at 60 °C and atmospheric pressure: pure water, 0.1 M KOH and 1.0 M KOH. Stability tests were conducted by applying a constant voltage of 2.2 V over an extended period of time. In addition to BoT and EoT characterization, the stability test was frequently interrupted by a characterization procedure to monitor performance changes over time. The recorded polarization curves and Nyquist plots obtained from EIS at 2.0 V are shown for pure water operation in **Figure** **3** and for KOH operation in Figure S1 (Supporting Information).The point in time of BoT is the same for all three AEMWE cells at 2 h, while the point in time of EoT differs. For pure water EoT is at 34 h of operation, for 0.1 M KOH at 58 h of operation and for 1.0 M KOH at 82 h of operation.

In the polarization curve, the current density serves as a measure of the hydrogen production rate and cell efficiency, while the cell voltage is the driving force of the electrolysis. For pure water operation (see Figure 3A), the polarization curves changed considerably over time. This is given by the curve shifting to lower current densities (and higher cell voltages) and by the high increase in the slope, indicating activation and ohmic losses reducing the hydrogen production rate and cell efficiency. Its deterioration occurred particularly between 10 and 34 h of operation that marks the EoT for the pure‐water‐fed AEMWE cell. In contrast, both KOH‐fed AEMWE cells exhibited higher current densities and less changes in the polarization curves (see Figure S1, Supporting Information).

**Figure 4:**
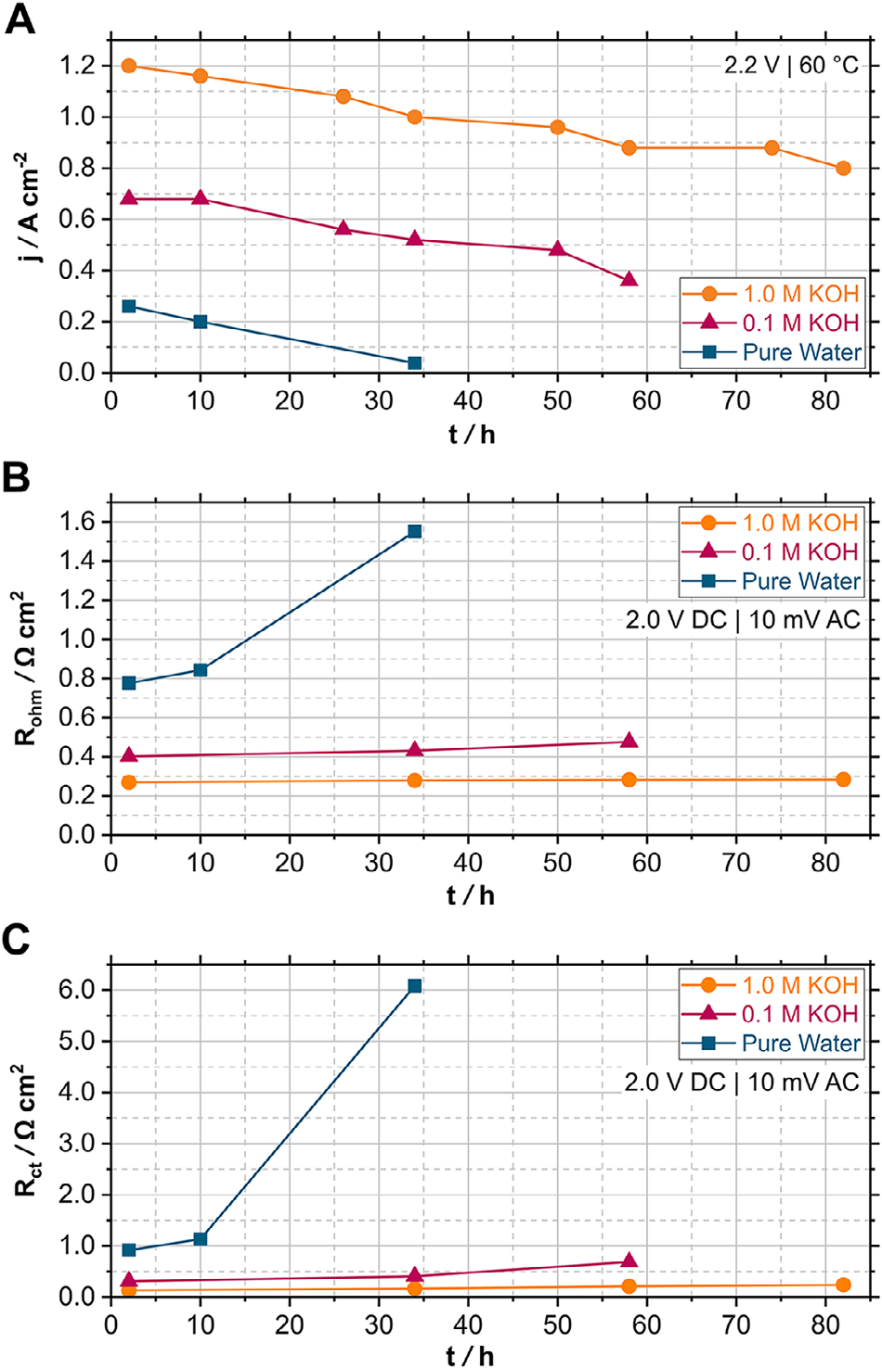
A) Time‐resolved change in current density *j* of the AEMWE cells at 2.2 V; time‐dependent change in B) ohmic resistance *R*_ohm_ and C) charge transfer resistance *R*_ct_, obtained from EIS at 2.0 V.

For better comparison and discussion of the changes occurring during operation, the current density at 2.2 V from the polarization curves is plotted over time in **Figure** **4**A for the MEAs operated in pure water, 0.1 M KOH and 1.0 M KOH. The pure‐water‐fed AEMWE cell delivered a current density of 0.26 A cm^−2^ at a voltage of 2.2 V at BoT which decreased to 0.20 A cm^−2^ after 10 h of operation and drastically declined to 0.04 A cm^−2^ at EoT. In contrast, the KOH‐fed AEMWE cells showed a lower decrease in current density and thus in cell efficiency. The values at BoT and EoT are given in **Table** **2**.Degradation rates were calculated by dividing the decreased current density by the operation time. This results in degradation rates of 7 mA cm^−2^ h^−1^, 6 mA cm^−2^ h^−1^ and 5 mA cm^−2^ h^−1^ for pure water, 0.1 M KOH and 1.0 M KOH respectively. Hence, the AEMWE cells show a decrease in cell efficiency over time, with the performance degradation being the lowest for 1.0 M KOH, intermediate for 0.1 M KOH and highest for pure water. This indicates that the concentration of hydroxide ions is a key factor in maintaining the cell efficiency.

**Table 2:** Degradation rates and change in current density *j* at 2.2 V from BoT to EoT for AEMWE cells operated in pure water, 0.1 M KOH and 1.0 M KOH.

	*j*_*BoT*_	*j*_*EoT*_	degradation rate
	/A cm^−2^	/A cm^−2^	/A cm^−2^ h^−1^
Pure Water	0.26	0.04	0.007
0.1 M KOH	0.68	0.36	0.006
1.0 M KOH	1.20	0.80	0.005

To identify underlying causes of the observed performance changes, EIS was employed. The Nyquist plots in Figure 3B and in Figure S1 (Supporting Information) visualize the increase of impedance, high frequency resistance (HFR), and low frequency resistance (LFR) over time for all three AEMWE cells, with the highest increase for the pure‐water‐fed AEMWE cell and the lowest increase for the one operated in 1.0 M KOH. This is in good agreement with the decrease in cell efficiency given by the polarization curves. HFR accounts for ohmic losses and is the intersection with the x‐axis at high frequencies (left‐hand side of the Nyquist plot). Accordingly, the intersection with the x‐axis at low frequencies (right‐hand side of the Nyquist plot) is the LFR that includes ohmic, activation, and mass transport losses.

To gain deeper insight, the EIS data was fitted using the equivalent circuit model introduced in our previous work.^[^ ^25^ ^]^ It consists of an inductor (*L*), an ohmic resistance (*R*_ohm_) and two resistor‐constant phase element (CPE) elements representing the activation resistances and double layer capacitance of anode (*R*_a_, *CPE*_a_) and cathode (*R*_c_, *CPE*_c_) (see Figure S2, Supporting Information). *R*_a_ and *R*_c_ are the kinetic resistances resulting from the activation energy required for the reactions at the respective electrode. They give insight into the condition of the electrode, particularly of the catalyst layer (CL), including the catalytic activity, the electrochemically active surface area (ECSA) and number of reaction sites. *R*_ohm_ equals the HFR and refers to the resistance encountered by the electric current when passing through the cell. It includes the inherent resistance of each material as well as the ionic and interface resistance of all cell components.

The obtained resistances at 2.0 V are presented in Figure 4B,C showing the time‐resolved trends of ohmic resistance *R*_ohm_ and charge transfer resistance *R*_ct_. Here, *R*_ct_ is the sum of the activation resistance of anode (*R*_a_) and cathode (*R*_c_). The values for *R*_ohm_ and *R*_ct_ at BoT and EoT are provided in **Table** **3**.The pure‐water‐fed MEA exhibited the highest increase in *R*_ohm_ and *R*_ct_ during operation. *R*_ohm_ was doubled, while *R*_ct_ showed a sixfold increase. In contrast, the MEA operated in 1.0 M KOH demonstrated only a slight increase in *R*_ohm_ and *R*_ct_ from BoT to EoT. *R*_ct_ was almost doubled, while *R*_ohm_ nearly stayed the same. In 0.1 M KOH, the time‐resolved trends of the resistances are more similar to the ones of 1.0 M KOH than to the ones of pure water. *R*_ohm_ slightly increased, while *R*_ct_ was more than doubled.

**Table 3:** Change in resistances *R*_ohm_ and *R*_ct_ at 2.0 V from BoT to EoT for AEMWE cells operated in pure water, 0.1 M KOH and 1.0 M KOH.

		*R*_ohm_/Ω cm^2^	*R*_ct_/Ω cm^2^
Pure Water	BoT	0.78	0.92
	EoT	1.55	6.08
0.1 M KOH	BoT	0.40	0.31
	EoT	0.48	0.69
1.0 M KOH	BoT	0.27	0.13
	EoT	0.28	0.24

These results confirm that the observed lower cell efficiency and stability of the pure‐water‐fed AEMWE cell (see Figures 3 and 4A) are caused by higher resistances *R*_ohm_ and *R*_ct_. This may result from a lower ionic conductivity, lower catalytic activity, lower catalyst utilization, lower number of reaction sites, and an increased degradation, due to a lower hydroxide ion concentration and lower pH compared to the KOH‐fed AEMWE cells.

Comparing the three different feeds, it is obvious that a low increase of hydroxide ion concentration, as it is the case for operation with 0.1 M KOH, has a considerable positive effect on the cell efficiency and stability of the AEMWE cell. *R*_ohm_ and *R*_ct_ of the AEMWE cell operated in 0.1 M KOH are reduced by half doubling the current density compared to the pure‐water‐fed one.

All three electrolysis tests show a higher increase in *R*_ct_ than in *R*_ohm_. This indicates that the observed efficiency loss is mainly caused by changes in the CL and interface of electrode and membrane. The increase in *R*_ct_ could be attributed to a decline in catalytic activity, a reduction of ECSA and a loss of reaction sites.

**Figure 5:**
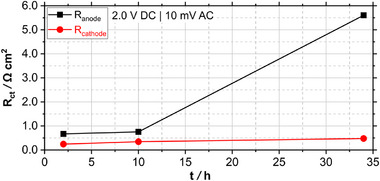
Time‐resolved change in charge transfer resistance *R*_ct_ of the pure‐water‐fed AEMWE cell, separated in the contributions of anode *R*_a_ and cathode *R*_c_, obtained from EIS at 2.0 V.

To differentiate and evaluate the contributions of anode and cathode to *R*_ct_, **Figure** **5** displays the activation resistances of anode *R*_a_ and cathode *R*_c_ for the pure‐water‐fed AEMWE cell. From our previous work on the BoT of the same AEMWE cell,^[^ ^25^ ^]^ it is expected that *R*_a_ is higher than *R*_c_, as the oxygen evolution reaction (OER) is the slower and more energy‐demanding reaction, due to the four‐electron process and the O_2_ formation, compared to the hydrogen evolution reaction (HER). Indeed, *R*_a_ is about three times higher at BoT and more than ten times higher at EoT compared to *R*_c_. From BoT to EoT, *R*_c_ was doubled, while *R*_a_ increased by a factor of ten, clearly showing a higher degradation of the anode. These results confirm a higher contribution of the anode to *R*_ct_ compared to the cathode.

Shifting the focus to the membrane, membrane thinning and short‐circuit were not detected in Figure 4 and in Figure S1 (Supporting Information). Membrane thinning can be revealed by a decrease of *R*_ohm_ that is not the case here. Some catalyst residues were visible on both sides of the membranes which were stamped by the flow fields and fibrous PTLs due to the contact pressure applied in the AEMWE cell (see Figure S3, Supporting Information). Still, the AEMs were not visibly damaged and no pinholes were evident upon cell disassembly after EoT, implying mechanical stability of the AEM. These findings suggest that mechanical and chemical degradation of the AEM may not have occurred during the measurement. This is in line with previously published work^[^ ^6^, ^26^, ^27^ ^]^ stating membrane degradation taking place after a longer period of time. Nevertheless, membrane degradation cannot be fully excluded for pure water operation, since a rapid decrease in cell performance and a significant increase of *R*_ohm_ were observed. In general, membrane degradation can be caused by insufficient mechanical and chemical stability of the AEM. Various chemical degradation mechanisms have been presented such as nucleophilic attack and Hofmann elimination.^[^ ^6^, ^26^, ^28^, ^29^ ^]^ All of these mechanisms provoke the deactivation and loss of functional groups, decreasing the ionic conductivity and increasing *R*_ohm_. The investigation of possible underlying membrane degradation mechanisms, including chemical analysis of the AEM to determine functional group integrity and chemical stability, is not trivial and beyond the scope of this study. However, it is assumed that severe membrane degradation did not take place and membrane degradation only played a minor part in pure water operation. The considerably higher increase of *R*_ct_ implies that the reasons for the increase of *R*_ohm_ are mainly related to electrode degradation and changes in the CL.

### 2.2 Morphological and Elemental Studies on the MEAs

To analyze structural changes and variations in material composition, scanning electron microscopy (SEM) and energy‐dispersive X‐ray spectroscopy (EDS) were applied on selected MEAs. Top‐view SEM images are shown of the pristine MEA as well as of the post mortem MEA operated in pure water and of the post mortem MEA operated in 1.0 M KOH. Cross‐sectional SEM images were taken of the pristine MEA and the post mortem MEA operated in 1.0 M KOH. It is assumed that the findings from the cross‐sectional analysis of the KOH‐fed MEA are transferable to the post mortem MEA operated in pure water.

**Figure 6:**
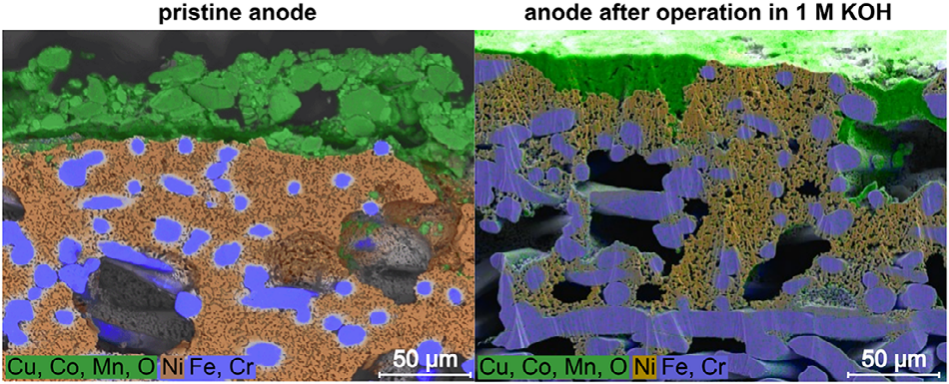
Cross‐sectional SEM images including EDS mapping of the Cu_0.6_Mn_0.3_Co_2.1_O_4_ anode at the pristine state (left) and at the post mortem state after operation in 1.0 M KOH (right). Quantitative EDS analysis can be found in Table S1 (Supporting Information). Higher‐resolution cross‐sectional SEM images (without EDS mapping) can be found in Figure S4 (Supporting Information).

The cross‐sectional SEM images including EDS mapping of the Cu_0.6_Mn_0.3_Co_2.1_O_4_ anode in **Figure** **6** show the pristine anode (left) and the post mortem anode operated in 1.0 M KOH (right).Prior to applying the catalyst, the stainless steel substrate was modified by depositing nickel to increase its surface for a higher catalyst utilization. The fine‐pored sponge‐like structure composed of nickel is located on and between the fibers in the upper part of the 316L‐fiber felt. The thickness of this layer ranges from 100 to 200μm in both the pristine and post mortem sample. On top of this nickel‐coated stainless steel felt is the CL. Looking at the CL of the pristine anode, catalyst particles are less homogeneously distributed on the substrate, whereas the CL of the anode operated in 1.0 M KOH is more densely packed with less voids. The pristine anode exhibits a CL thickness of 10 to 40μm, while the CL thickness of the post mortem anode is 1 to 20μm. Reasons for the decrease of CL thickness are the compression of the MEA in the cell housing as well as the loss of catalyst and ionomer in the CL. (Catalyst and ionomer loss are confirmed and further discussed below.) When discussing changes in the MEA ex situ, it is important to consider the damage and changes that occurred due to the assembly and disassembly of the cell for electrolyzer tests. Hence, the decrease of CL thickness is mainly caused by the compression of the MEA when installed in the cell housing. This led to the compression of the CL and also forced catalyst into the pores of the PTL. Additionally, parts of the CL remained attached to the membrane and are therefore not visible in the SEM images of the electrodes. This was evident when the cell and MEA were disassembled (see Figure S3, Supporting Information). To identify the contributions of mechanical compression and electrochemical degradation, future work should analyze pristine electrodes after cell assembly and compression but before electrochemical testing as an intermediate reference state.

**Figure 7:**
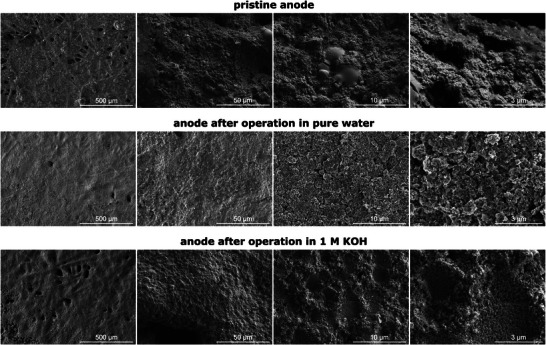
Top‐view SEM images with different magnifications of the Cu_0.6_Mn_0.3_Co_2.1_O_4_ anode at the pristine state (top row) as well as at the post mortem state after operation in pure water (middle row) and in 1.0 M KOH (bottom row). Higher‐resolution SEM images can be found in Figure S5 (Supporting Information).

**Figure** **7** shows the top‐view SEM images of the non‐precious metal anode at the pristine state (top row) as well as at the post mortem state after operation in pure water (middle row) and after operation in 1.0 M KOH (bottom row).The stainless steel fibers of all three anodes are covered with nickel and catalyst Cu_0.6_Mn_0.3_Co_2.1_O_4_ forming a closed CL with a few gaps between the fibers (see Figure 7, left column (500 µm)). For the pure‐water‐fed anode, there are hardly any gaps at the interface of anode and membrane. This may negatively impact the oxygen gas removal, as the gas bubbles might get trapped resulting in an increased mass transport resistance which is evident in the Nyquist plot in Figure 3B. Trapped oxygen gas bubbles may also block reaction sites leading to an increased *R*_ct_ and thus a lower cell performance compared to BoT and to the KOH‐fed AEMWE cell.

Comparing the three anodes, the pristine anode exhibits a more inhomogeneous surface with large oxide particle agglomerates of up to 100μm that are not present for the electrochemically tested ones. Indeed, the post mortem anodes are composed of smaller particles (40 to 100 nm) and agglomerates (up to 1μm) indicating a restructuring of the CL (see Figure 7). This fragmentation of larger catalyst particles into smaller ones formed a more uniformly distributed layer of small particles compared to the pristine anode. This significant morphology change for CoO, when electrochemically oxidized in alkaline media, has also been observed by other scholars.^[^ ^30^, ^31^ ^]^

Comparing the anode catalyst particles at the post mortem states (see Figure 7, right column (3μm)), the pure‐water‐fed anode has a more flake‐like structure. In contrast, the KOH‐fed anode exhibits a more granular structure and smaller catalyst particles. This indicates a larger ECSA and a larger number of reaction sites in the KOH‐fed anode that (partly) explains the lower *R*_ct_ and higher cell efficiency for KOH operation compared to pure water operation.

Furthermore, both post mortem anodes show a roughening of the CL compared to the pristine one. This can be attributed to a loss of ionomer and catalyst in the CL, as described by other research groups.^[^ ^26^, ^31^, ^32^, ^33^, ^34^ ^]^ Indeed, EDS elemental analysis of cross‐section and top view (see Tables S1 and S2, Supporting Information) revealed a relative reduction of the ionomer content in the CL of both post mortem anodes, implying ionomer loss. This is supported by X‐ray photoelectron spectroscopy (XPS) displaying a reduction of the nitrogen content in the CL of the KOH‐fed anode and no detectable nitrogen content in the CL of the pure‐water‐fed anode (see Figure S6 and Table S3, Supporting Information). Here, nitrogen is a marker for the C‐N bonds in the quaternary ammonium groups of the ionomer. The degradation and loss of ionomer, due to ionomer poisoning, ionomer oxidation, and ionomer detachment, has been reported as a main reason for degradation and performance loss in AEMWE.^[^ ^9^, ^18^, ^27^, ^32^, ^33^ ^]^ This occurs particularly under the harsh conditions of OER and is accelerated under high cell voltage (> 2.0 V) in alkaline media.^[^ ^9^, ^18^, ^33^ ^]^

To understand the impact of ionomer degradation on the cell performance and its deterioration, the critical roles of the ionomer in AEMWE systems and the importance for pure water operation are discussed in the following paragraphs. The ionomer in the CL provides pathways for hydroxide ions between membrane and catalyst which is crucial for the ionic conductivity (*R*_ohm_) and the supply of hydroxide ions for the OER (*R*_ct_). It also serves as a binder in the electrode, connecting catalyst particles and adhering catalyst to the substrate, providing mechanical support.^[^ ^35^, ^36^, ^37^ ^]^ Thus, ionomer loss can lead to catalyst detachment and catalyst loss, which may result in a decrease of ECSA and reaction sites, increasing *R*_ct_. It may also lead to a lower electrical conductivity increasing *R*_ohm_, since the catalyst takes part in conducting electrons from the reactions sites in the CL to the PTL.

**Figure 8:**
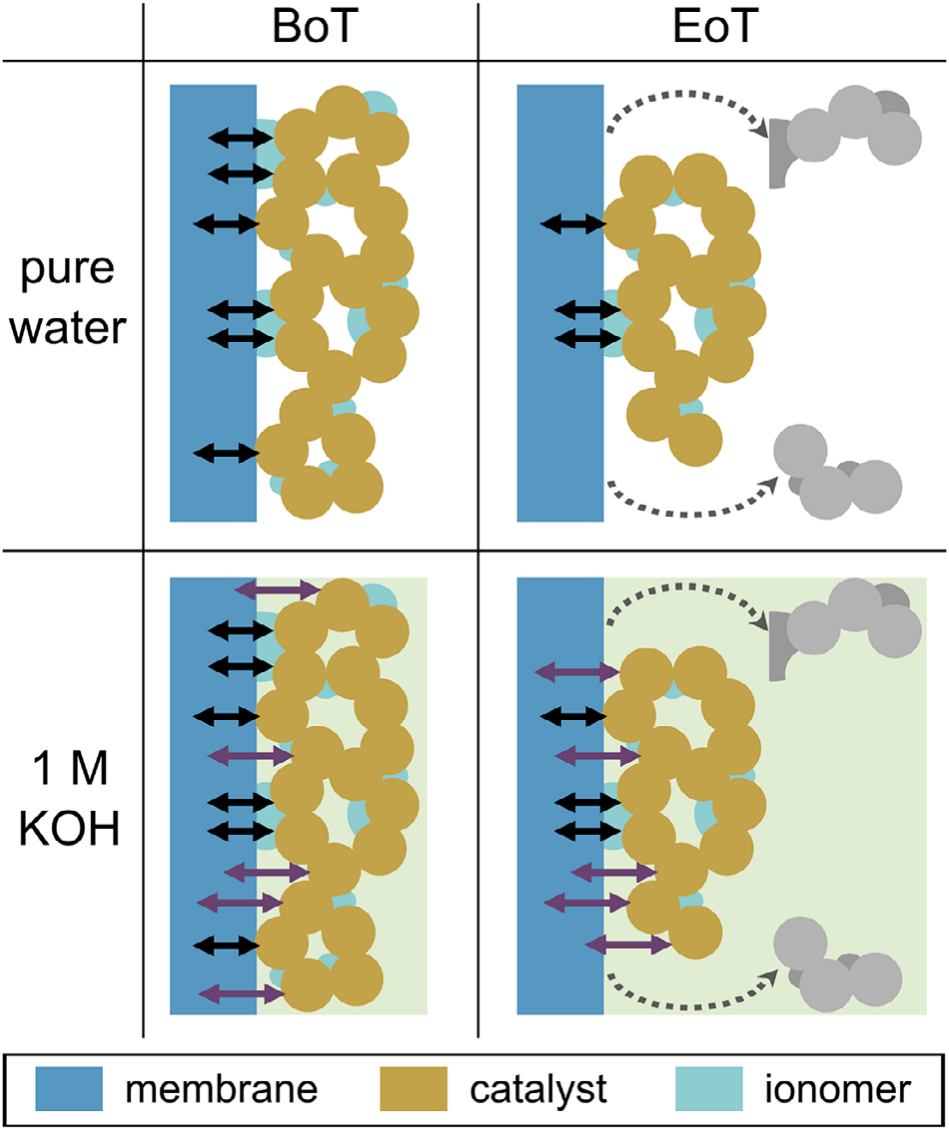
Schematic representation of the hydroxide ion transport at the interface of membrane and electrode at BoT (left) and EoT (right) when the AEMWE cell is operated with pure water (top row) and aqueous KOH (bottom row). Black arrows represent the ion pathways between AEM and catalyst in direct contact and in contact through ionomer. Purple arrows illustrate additional ion pathways through the supporting liquid electrolyte. Grey objects represent the loss of catalyst and ionomer.

As visible in Figure 7, the CL of the pure‐water‐fed anode is less connected and more coarse‐grained than the KOH‐fed one. There appears to be less ionomer present on the surface of the pure‐water‐fed anode, indicating a higher ionomer loss in pure water. Additionally, the ionomer plays a more important role in pure water operation than in KOH operation, as schematized in **Figure** **8**. In pure water, the hydroxide ion transfer between AEM and catalyst only occurs through direct contact of the membrane and the catalyst particles and through contact of the membrane and the ionomer that is connected to catalyst particles. However, in aqueous KOH, additional hydroxide ion pathways are provided. This allows reactions to occur on catalyst surfaces that are not in contact with ionomer, resulting in more reaction sites and a higher catalyst utilization. Hence, if ionomer loss takes place, it affects more the pure‐water‐fed electrolyzer, as visualized in Figure 8, resulting in a lower hydroxide ion conductivity and in catalyst detachment and loss of reaction sites. This may explain the higher increase of *R*_ohm_ and *R*_ct_ and consequently higher cell efficiency losses over time in pure water operation compared to KOH operation, where the supporting liquid electrolyte may conceal the ionomer loss by providing hydroxide ions. This is consistent with *R*_ohm_ remaining the same in 1.0 M KOH.

To fully elucidate the underlying degradation mechanisms, additional advanced characterization methods are required that are beyond the scope of the present study. Comprehensive ionomer analysis and quantification of changes in ionomer coverage, catalyst particle size distribution, and porosity would necessitate sample embedding combined with advanced imaging techniques such as focused ion beam scanning electron microscopy (FIB‐SEM), scanning transmission electron microscopy (STEM) or high‐resolution transmission electron microscopy (HRTEM) and statistical evaluation of multiple non‐adjacent regions for representative sampling. It would also benefit from direct chemical evidence of ionomer changes via Fourier transform infrared (FTIR) spectroscopy to analyze functional group integrity and via detailed core‐level XPS spectra of C and N. Additionally, catalyst degradation mechanisms would be more thoroughly resolved through X‐ray diffraction (XRD) and detailed core‐level XPS of the catalyst, identifying phase transitions and elemental valence changes. Future work should incorporate these complementary techniques to enable more definitive mechanistic conclusions.

**Figure 9:**
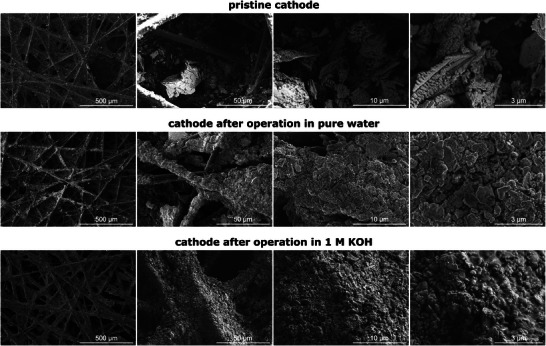
Top‐view SEM images with different magnifications of the Raney‐Nickel cathode at the pristine state (top row) as well as at the post mortem state after operation in pure water (middle row) and in 1.0 M KOH (bottom row). Higher‐resolution SEM images can be found in Figure S7 (Supporting Information).

Shifting the focus to the cathode, **Figure** **9** presents the top‐view SEM images of the Raney‐Nickel cathode at the pristine state (top row) as well as at the post mortem state after operation in pure water (middle row) and after operation in 1.0 M KOH (bottom row).In contrast to the anode, the cathode catalyst does not form a closed layer, as the carbon PTL is only partly covered with catalyst. Catalyst particles and agglomerates are less homogeneously distributed on the PTL of the pristine cathode compared to the post mortem cathodes. The cathode operated in 1.0 M KOH exhibits full catalyst coverage of the PTL fibers, while the PTL fibers of the pure‐water‐fed cathode are partly covered. This considerable change compared to the pristine cathode can be related to a restructuring of the CL and change in composition during HER.

**Figure 10:**
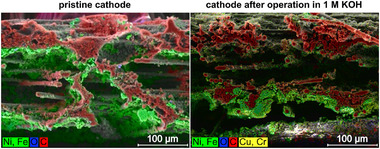
Cross‐sectional SEM images including EDS mapping of the Raney‐Nickel cathode at the pristine state (left) and at the post mortem state after operation in 1.0 M KOH (right). Quantitative EDS analysis can be found in Table S4 (Supporting Information). Higher‐resolution cross‐sectional SEM images (without EDS mapping) can be found in Figure S8 (Supporting Information).

Indeed, the cross‐sectional SEM‐EDS images of the cathode in **Figure** **10** and the EDS analysis show a higher amount of oxygen in the CL of both post mortem cathodes, detected by higher blue contents in the CL (see Figure 10(right)) and supported by EDS quantification (see Tables S4 and S5, Supporting Information). This impacts the HER activity increasing *R*_ct_ and may also lead to a higher *R*_ohm_ due to a higher interface resistance and a higher electrical resistance in the cathode. A main degradation phenomenon for nickel as cathode catalyst has been reported to be hydride formation, decreasing the HER activity.^[^ ^28^, ^38^, ^39^ ^]^ It occurs when oxygen from the electrolyte is adsorbed at the surface of metallic nickel, forming NiO and transforming into NiOOH.^[^ ^38^, ^39^ ^]^ As the formation of an oxygen‐rich CL during HER is confirmed by Figure 10 and the increase of *R*_ct_ is given by Figure 4C, we conclude that hydride formation may be a cause for the observed changes in the cathode. To fully describe and understand the underlying mechanisms, additional characterization methods, such as XRD and detailed core‐level XPS of the catalyst, are necessary that are beyond the scope of this study.

Regarding the cross‐sectional distribution of the cathode (see Figure 10), the catalyst is not only deposited on the surface of the PTL (the interface to the membrane), but extends over the entire cross section of the PTL. Consequently, there is a larger transport distance for the hydroxide ions from the reaction sites in the cathode to the membrane, decreasing the cell efficiency. This can be (partly) compensated by a high ionic conductivity, for example due to a higher concentration of hydroxide ions from a supporting electrolyte, as it is the case for KOH operation. However, the top‐view SEM images of the pure‐water‐fed cathode (see Figure 9 (middle row)) show a rougher, less‐connected CL and less ionomer present on the cathode surface compared to the KOH‐fed one (see Figure 9 (bottom row)). This is supported by XPS revealing a reduction of the nitrogen content in the CL of both post mortem cathodes and a lower nitrogen content in the pure‐water‐fed compared to the KOH‐fed one (see Figure S9 and Table S6, Supporting Information). These findings indicate ionomer loss resulting in a lower ionic conductivity in the pure‐water‐fed cathode. Therefore, the catalyst inside the PTL may not be (fully) utilized and may even impede the conduction of electrons.

Furthermore, elements such as Cu and Cr appear in the EDS mapping in the cross‐sectional SEM image after operation in 1.0 M KOH (see Figure 10 (right)) that are not visible in the pristine cathode (see Figure 10 (left)). This is supported by EDS analysis of anode and cathode, detecting Cu and Cr in both post mortem cathodes but not in the pristine cathode (see Tables S4 and S5, Supporting Information) as well as displaying a relative reduction of Cu content in the CL of both post mortem anodes (see Tables S1 and S2, Supporting Information). Additionally, Cu and Cr dissolution are confirmed by inductively coupled plasma mass spectrometry (ICP–MS) analysis of the electrolyte after AEMWE single cell operation (see Table S7, Supporting Information). These findings imply the dissolution of elements from the catalyst (Cu) and PTL (Cr) of the anode, their mitigation through the MEA and their redeposition in the cathode. This is in agreement with published work on CuCoO_*x*_ catalysts, observing a slight dissolution of Cu from the OER catalyst, particularly in neutral media and at open circuit voltage (OCV).^[^ ^37^, ^40^, ^41^ ^]^ As our test protocol included EIS and polarization curve step at OCV, the potential for Cu dissolution was present. Furthermore, it has been reported that stainless‐steel PTLs undergo corrosion for operation above 2.0 V.^[^ ^9^ ^]^ Since this is the case here, this may be a reason for the observed Cr dissolution. Element dissolution can lead to blocked reaction sites and ionomer poisoning when redeposited in the electrodes, increasing *R*_ct_ and *R*_ohm_, as described in previously published studies,^[^ ^33^, ^39^, ^42^ ^]^ impacting the performance and durability of the AEMWE cell.

### 2.3 Approaches to Improve Cell Efficiency and Stability

The electrochemical and morphological evaluation revealed that ionomer degradation, catalyst loss, element dissolution and mass transport limitation are major causes for the observed decreased cell efficiency and stability of the AEMWE cells, particularly with pure water feed. Approaches to inhibit and reduce these degradation issues are mainly related to electrode and interface engineering. However, adjusting operating parameters, such as limiting the maximum cell voltage, can also have a significant effect. As most degradation mechanisms occur or are accelerated at high cell voltages, it is suggested to limit the operation of AEMWE to a maximum cell voltage of 2.0 V to reduce and prevent ionomer degradation, element dissolution and PTL corrosion.

Preventing ionomer degradation is of particular importance, since the ionomer plays a critical role in pure water operation and its degradation also leads to catalyst loss. To reduce ionomer degradation, most approaches focus on ionomer optimization, including developing advanced ionomers,^[^ ^21^ ^]^ selecting appropriate ionomers^[^ ^16^, ^27^, ^35^, ^43^ ^]^ as well as optimizing ionomer content and distribution.^[^ ^27^, ^35^, ^36^, ^43^, ^44^, ^45^, ^46^, ^47^, ^48^, ^49^ ^]^ However, most of these studies utilized an additional liquid electrolyte and did not investigate the effect of their proposed ionomer optimization on pure‐water‐fed AEMWE. Regarding the oxidative instability of ionomers at the anode, Kwak et al. introduced an electron‐blocking but ion‐permeable passivation coating separating catalyst and ionomer to suppress ionomer oxidation and to increase CL stability in pure water operation.^[^ ^10^ ^]^

Furthermore, high catalyst utilization is crucial for maintaining the cell efficiency of pure‐water‐fed AEMWE. In addition to catalyst loading^[^ ^49^, ^50^ ^]^ and ionomer content, catalyst utilization also depends on catalyst adhesion and catalyst distribution, which are influenced by the deposition technique. Here, we used the hand‐spray airbrush method, while other studies demonstrated higher catalyst utilization, less catalyst loss, and increased CL stability using air‐plasma spraying,^[^ ^51^, ^52^, ^53^ ^]^ automated ultrasonic spray coating,^[^ ^31^, ^54^ ^]^ or direct bar coating.^[^ ^55^ ^]^ This indicates that using a different deposition technique may have reduced catalyst loss in our AEMWE cells and increased catalyst utilization and CL stability.

To enhance mass transport, one approach is to develop an electrode or PTL with a more optimal mixture of small and large pores to facilitate faster bubble detachment and promote gas removal. This, for example, was achieved by applying a microporous layer onto the PTL, as presented by Razmjooei et al.,^[^ ^52^ ^]^ or by using a double‐layered substrate fabricated by an overlaying strategy, as demonstrated by Ding et al.^[^ ^56^ ^]^ Another approach, reported by Wan et al.,^[^ ^15^, ^57^ ^]^ was the design of a three‐dimensionally‐ordered MEA with vertical channels that functioned as mass transfer highways in the CLs, AEM and at their interfaces.

### 2.4 Comparison with Literature and Comparison of Different Cell Sizes

**Figure 11:**
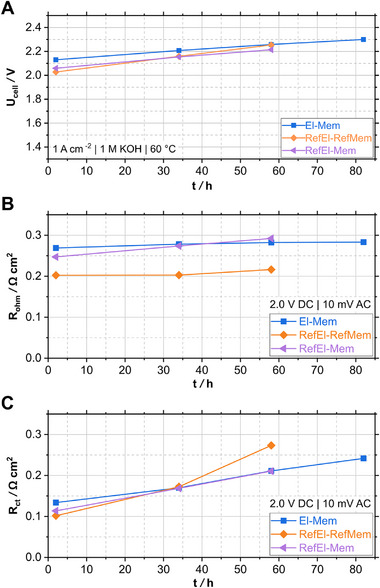
A) Time‐resolved change in current density *j* at 2.2 V of the MEAs El‐Mem, RefEl‐RefMem and RefEl‐Mem operated in 1.0 M KOH; time‐dependent change in B) ohmic resistance *R*_ohm_ and C) charge transfer resistance *R*_ct_, obtained from EIS at 2.0 V. The corresponding polarization curves and Nyquist plots can be found in Figure S10 (Supporting Information). (It is noted that stability tests of the MEAs RefEl‐RefMem and RefEl‐Mem could not be carried out in pure water due to the low catalytic activity of the commercial electrodes in pure water. BoT data of these MEAs in pure water can be found in our previous work.^[^ ^25^ ^]^)

Comparing results to existing literature can be challenging, primarily due to the lack of standardized testing in AEMWE and thus differences in test setups, protocols and operating conditions.^[^ ^58^, ^59^ ^]^ There is also a scarcity of studies on AEMWE tests performed in pure water, without a supporting electrolyte, utilizing 25 cm^2^ non‐precious metal electrodes. To address this, we carried out electrolysis tests using commercially available membranes and electrodes for comparison. Hence, three different MEAs were assembled and investigated: the developed membrane combined with the developed electrodes (MEA El‐Mem), the commercial membrane paired with the commercial electrodes (MEA RefEl‐RefMem) and the developed membrane in combination with the commercial electrodes (MEA RefEl‐Mem). It is noted that the membrane thickness of the developed and commercial AEM is the same (50μm), while the thickness of the developed electrodes is higher compared to the commercial ones (see Experimental Section 4). The measurements were performed in pure water and in 1.0 M KOH. However, due to the low catalytic activity of the commercial electrodes in pure water, stability tests of the MEAs RefEl‐RefMem and RefEl‐Mem could not be carried out in pure water. For these two MEAs, polarization curves and impedance spectra are only available for BoT, which were shown and compared to the MEA El‐Mem in our previous work.^[^ ^25^ ^]^ Therefore, solely the results of the AEMWE cells operated in 1.0 M KOH are presented in **Figure** **11** and in Figure S10 (Supporting Information). Stability tests were conducted by applying a constant current density of 1 A cm^−2^.

All three MEAs show a performance decrease over time, given by the increase in cell voltage, *R*_ohm_ and *R*_ct_ from BoT to EoT displayed in Figure 11 (the corresponding polarization curves and Nyquist plots can be found in Figure S10, Supporting Information). Although the fully commercial MEA RefEl‐RefMem attained the lowest cell voltage (2.03 V) at 1 A cm^−2^ at BoT, the cell voltage increased the most during the 58 h of operation with a degradation rate of 3.90 mV h^−1^. In contrast, the developed MEA El‐Mem, requiring the highest cell voltage (2.13 V) at 1 A cm^−2^ at BoT, had the lowest degradation rate with 2.21 mV h^−1^ at 58 h of operation. This indicates that the developed MEA El‐Mem can compete with the commercial one. It also underlines the importance of conducting stability tests, since the long‐term performance can differ from the initial performance.

Moreover, the commercial MEA RefEl‐RefMem exhibited a lower cell efficiency and stability compared to data of the same MEA type stated by the manufacturer and literature. Liu et al. reported a cell performance of 1.9 V at 1 A cm^−2^ and a degradation rate of 0.005 mV h^−1^ for 2000 h of operation.^[^ ^60^ ^]^ An even longer operating time was demonstrated with this commercial MEA type by Motealleh et al.^[^ ^12^ ^]^ They accomplished a long‐term test of 10000 h, yielding a degradation rate of 0.001 mV h^−1^ at 1 A cm^−2^. These lower degradation rates are related to lower *R*_ohm_ being 0.045 Ω cm^2^^[^ ^60^ ^]^ and 0.06 Ω cm^2^,^[^ ^12^ ^]^ while we achieved a *R*_ohm_ of 0.2 Ω cm^2^. *R*_ohm_ contains the electrical, contact and ionic resistances of all cell components and is also influenced by flow field design, MEA tightness and material conductivities.^[^ ^61^ ^]^ As the MEA type is the same, reasons may be differences in the test setup, cell size, material batch, test protocol, and operating parameters. Indeed, they used a 5 cm^2^ cell, while we utilized a larger AEMWE cell with an electrode area of 25 cm^2^. Another reason for the low degradation rates may be the long operating time. Some studies have shown that the increase in cell voltage, and therefore degradation, is highest in the first few hundred hours of operation, after which the cell voltage approaches a more constant level.^[^ ^13^, ^18^, ^21^, ^27^ ^]^ Our work falls within this range of high degradation operation time, whereas the two studies mentioned above greatly exceeded it. Consequently, conducting longer electrolysis tests may have reduced the degradation rates of our experiments. This hypothesis can be observed for the MEA El‐Mem operated in 1.0 M KOH for 84 h, delivering degradation rates of 2.21 mV h^−1^ at 58 h and a lower rate of 2.07 mV h^−1^ at 82 h.

To further contextualize the electrochemical performance of the 25 cm^2^ AEMWE cell with the developed MEA, a comparison with a smaller 5 cm^2^ cell was conducted (see Figure S11, Supporting Information). The results reveal differences in performance based on cell size and electrolyte feed. When operated with pure water, the 5 cm^2^ cell demonstrated a higher cell efficiency with lower *R*_ohm_ and lower *R*_ct_ compared to the 25 cm^2^ cell. While the resistances increased more significantly over time for the 25 cm^2^ cell, the degradation rates of both AEMWE cells were comparable (6.5 mA cm^−2^ h^−1^ at 34 h). However, when operated with 1.0 M KOH, the trends are different. The 25 cm^2^ cell delivered a higher current density, lower *R*_ohm_ and lower *R*_ct_, outperforming the 5 cm^2^ cell. In this case, although the resistances increased more significantly over time for the 5 cm^2^ cell, its degradation rate is somewhat lower compared to the one of the 25 cm^2^ cell (3.5 mA cm^−2^ h^−1^ vs. 4.9 mA cm^−2^ h^−1^ at 82 h). This comparison underscores the critical importance of experimental validation across multiple cell sizes, as performance and stability cannot be reliably predicted or extrapolated from one cell size to another. Therefore, conclusions drawn from small‐scale laboratory results (⩽5 cm^2^) might be misleading for technology assessment, as performance and degradation behavior can vary with scale. These findings highlight the necessity of evaluating large cell sizes to gain a more comprehensive understanding of the behavior and scalability of AEMWE technology for industrial application.

## 3 Conclusion

In this study, we investigated the degradation behavior of a pure‐water‐fed non‐precious metal 25 cm^2^ AEMWE cell in a short‐term durability test accompanied by SEM and EDS. Stability test and polarization curves showed that the AEMWE cell operated in pure water experienced high degradation, exhibiting lower cell efficiency and stability compared to KOH‐fed AEMWE cells. Reasons for this, confirmed by EIS analysis, were the increase of the charge transfer resistance *R*_ct_ and ohmic resistance *R*_ohm_, with the activation resistance at the anode *R*_a_ increasing the most. This was attributed to a loss of reaction sites and a reduction of ECSA. The electrochemical evaluation indicated that the deterioration of the AEMWE cell performance was mainly caused by electrode and interface degradation, particularly at the anode, rather than by membrane degradation. SEM and EDS analyses revealed significant changes in the morphology and elemental composition of the MEA after operation, such as restructuring of the CL and element dissolution. Integrating the results of the electrochemical and structural evaluation, ionomer degradation, impacting ionic conductivity, and mechanical stability of the CL, was found to be the key contributor to the observed reduced cell efficiency and stability. This was more pronounced for pure water operation compared to KOH operation, where the supporting liquid electrolyte provided a higher hydroxide ion concentration and additional reaction sites, masking ionomer loss. To advance AEMWE technology, it is suggested to increase the stability of pure‐water‐fed AEMWE by improving ionomers and the membrane‐electrode interface for pure water operation. Our work is expected to contribute to the development of more efficient and stable AEMWE systems for industrial application.

## 4 Experimental Section

### Membrane Electrode Assemblies

The membrane synthesized by Fumatech BWT GmbH was a polyaliphatic anion exchange membrane with a thickness of approximately 50μm. Prior to testing, the AEM underwent ion exchange by soaking in 1.0 M KOH for 24 h, with the solution refreshed after 8 h, followed by rinsing with ultra pure water ($0.055\;\mu S{\mathrm{cm}}^{-1}$).

The non‐precious metal electrodes were fabricated by Fraunhofer IFAM Dresden using the airbrush technique. As cathode, Raney‐Nickel, purchased from Gaskatel GmbH, was hand‐sprayed on carbon paper 36AA from SGL Carbon SE. As anode, 316L‐fiber felt from Bekaert was coated with nickel powder prior to applying the OER catalyst. The synthesis of the OER catalyst Cu_0.6_Mn_0.3_Co_2.1_O_4_ was based on a method adapted from Paknahad et al.,^[^ ^62^ ^]^ with additional details available in the previous work.^[^ ^25^ ^]^ These catalysts were selected in a pre‐screening evaluation within the project due to their activity in both electrolysis (OER and HER) and fuel cell (ORR and HOR) modes. The catalyst inks consisted of the respective catalyst, FAA3 ionomer from Fumatech BWT GmbH (20% ionomer content in relation to the sum of catalyst and ionomer weight) and n‐propanol as solvent. The catalyst loading was estimated at 4 mg cm^−2^, determined through mass differential analysis before and after catalyst application on the porous transport layer (PTL). Electrode thickness was approximately 750μm for the anode and 250μm for the cathode.

For comparison to commercially available materials, electrodes, and membranes were sourced from Dioxide Materials, using Sustainion®X37‐50 Grade RT as AEM (50μm), NiFe_2_O_4_ on stainless steel fiber paper as anode (600μm) and NiFeCo on nickel fiber paper as cathode (300μm).^[^ ^60^ ^]^

### AEMWE Test‐Setup

All AEMWE tests were carried out using an in‐house built test bench. Electrochemical measurements were performed with the Gamry Reference 3000 potentiostat/galvanostat and the Gamry Reference 30k booster. The MEAs were assembled with PTFE gaskets in a 25 cm^2^ electrolyzer test cell commercially available from Dioxide Materials, consisting of flow field and end plates made of titanium and stainless steel. For the comparison of different cell sizes, a 5 cm^2^ test cell with Nickel plates was used. Once the electrolyzer cell was installed in the test bench, the system was preheated for 30 min circulating pure water or aqueous KOH. All electrolysis tests were conducted at 60°C under atmospheric pressure, with the cell temperature monitored via a thermocouple placed in the anode flow field plate. Pure water ($0.055\;\mu S{\mathrm{cm}}^{-1}$), 0.1 M KOH (pH 13) or 1.0 M KOH (pH 14) were used to supply the anode and cathode with flow rates of 80 mL min^−1^ for the 25 cm^2^ cell and 50 mL min^−1^ for the 5 cm^2^ cell.

### Electrochemical Characterization

After an activation at cell voltages of 1.8 and 2.2 V for one hour, polarization curves and electrochemical impedance spectroscopy (EIS) were recorded to characterize the initial performance of the AEMWE cells. Stability tests were carried out at 2.2 V, when comparing pure water feed and KOH feed, or at 1 A cm^−2^, when comparing with commercial materials in 1.0 M KOH. These tests were interrupted by polarization curves and EIS to evaluate performance changes. Polarization curves were obtained by incrementally increasing the current density until reaching a maximum cell voltage of 2.4 V, with each step maintained for at least 2 min. EIS was performed from 100 kHz to 100 mHz, capturing ten points per decade, and was conducted at 0.01 A cm^−2^, 1.8, 2.0. and 2.2 V, with amplitudes of 0.005 A cm^−2^ and 0.01 V. respectively. For KOH‐fed tests, EIS measurements were also taken at 0.1 and 0.2 A cm^−2^ with a 0.05 A cm^−2^ amplitude, maintaining the respective voltage or current density for 5 min before the measurement.

### Physical and Chemical Characterization

Cross‐sections of samples were prepared by Ar^+^ ion‐beam milling with LN_2_ cooling with a Gatan Ilion^+^ II device.

Top‐view scanning electron microscopy (SEM) was performed using a Hitachi SU70 device at an accelerating voltage of 3 kV with a working distance of 9.3 to 10.5 mm. Secondary electron (SE) images, in particular SE(L) and SE(M), are shown in this work.

Cross‐sectional SEM and energy dispersive X‐ray spectroscopy (EDS) were carried out using a Zeiss Sigma 300 device equipped with a peltier cooled Oxford UltimMax40 EDS detector and usage of 3 kV acceleration voltage for SEM and 20 kV acceleration voltage for EDS, a 120μm aperture in high current mode, a working distance of 8.5 mm, vacuum of less than 10^−6^ mbar and 20 keV detector range. Acquisition and quantification of measured data was done using Oxfords AZtec 6.1 software based on factory quantification standards.

A Thermo Fisher XPS Nexsa G2 photoelectron spectrometer (1486.6 eV) was used for X‐ray photoelectron spectroscopy (XPS). The spectra were obtained between 1350 and −10 eV with a step size of 0.5 eV, analyzer pass energy of 200 eV and 50 ms dwell time per energy set point.

For the electrolyte element analysis, inductively coupled plasma mass spectrometry (ICP–MS) was performed on an Agilent 8900 Triple Quadrupole device. Each electrolyte sample was neutralized with nitric acid and diluted. Three replicates of each sample were analyzed and the results were averaged.

## Conflict of Interest

The authors declare no conflict of interest.

## Supporting information



Supporting Information

## Data Availability

The data that support the findings of this study are available from the corresponding author upon reasonable request.

## References

[1] K. Ayers , N. Danilovic , R. Ouimet , M. Carmo , B. Pivovar , M. Bornstein , Annu. Rev. Chem. Biomol. Eng. 2019, 10, 219. 31173524 10.1146/annurev-chembioeng-060718-030241

[2] IEA ‐ International Energy Agency Global hydrogen review 2023, can be found under https://www.iea.org/reports/global‐hydrogen‐review‐2023, (accessed: November 2025).

[3] M. Chatenet , B. G. Pollet , D. R. Dekel , F. Dionigi , J. Deseure , P. Millet , R. D. Braatz , M. Z. Bazant , M. Eikerling , I. Staffell , P. Balcombe , Y. Shao‐Horn , H. Schäfer , Chem. Soc. Rev. 2022, 51, 4583. 35575644 10.1039/d0cs01079k PMC9332215

[4] H. A. Miller , K. Bouzek , J. Hnat , S. Loos , C. I. Bernäcker , T. Weißgärber , L. Röntzsch , J. Meier‐Haack , Sustainable Energy Fuels 2020, 4, 2114.

[5] I. Vincent , D. Bessarabov , Renewable Sustainable Energy Rev. 2018, 81, 1690.

[6] D. Henkensmeier , M. Najibah , C. Harms , J. Žitka , J. Hnát , K. Bouzek , J. Electrochem. Energy Convers. Storage 2021, 18, 2.

[7] S. Peng , Electrochemical Hydrogen Production from Water Splitting: Basic, Materials and Progress, Springer Nature Singapore and Imprint, 1st ed. 2023 edition, Springer, Singapore 2023.

[8] C. Li , J.‐B. Baek , Nano Energy 2021, 87, 106162.

[9] D. Li , A. R. Motz , C. Bae , C. Fujimoto , G. Yang , F.‐Y. Zhang , K. E. Ayers , Y. S. Kim , Energy Environ. Sci. 2021, 14, 3393.

[10] M. Kwak , K. Ojha , M. Shen , S. W. Boettcher , ACS Energy Lett. 2024, 9, 1025.

[11] Clean Hydrogen Partnership, Clean hydrogen joint undertaking strategic research and innovation agenda 2021‐2027, can be found under https://www.clean‐hydrogen.europa.eu/knowledge‐management/strategy‐map‐and‐key‐performance‐indicators/clean‐hydrogen‐ju‐sria‐key‐performance‐indicators‐kpis, (accessed: November 2025).

[12] B. Motealleh , Z. Liu , R. I. Masel , J. P. Sculley , Z. Richard Ni , L. Meroueh , Int. J. Hydrogen Energy 2021, 46, 3379.

[13] M. Moreno‐González , P. Mardle , S. Zhu , B. Gholamkhass , S. Jones , N. Chen , B. Britton , S. Holdcroft , J. Power Sources Adv. 2023, 19, 100109.

[14] N. Chen , S. Y. Paek , J. Y. Lee , J. H. Park , S. Y. Lee , Y. M. Lee , Energy Environ. Sci. 2021, 14, 6338.

[15] L. Wan , Z. Xu , Q. Xu , P. Wang , B. Wang , Energy Environ. Sci. 2022, 15, 1882.

[16] Y. Zheng , A. Serban , H. Zhang , N. Chen , F. Song , X. Hu , ACS Energy Lett. 2023, 8, 5018.

[17] G. A. Lindquist , S. Z. Oener , R. Krivina , A. R. Motz , A. Keane , C. Capuano , K. E. Ayers , S. W. Boettcher , ACS Appl. Mater. Interfaces 2021, 13, 51917. 34374278 10.1021/acsami.1c06053

[18] G. A. Lindquist , J. C. Gaitor , W. L. Thompson , V. Brogden , K. J. T. Noonan , S. W. Boettcher , Energy Environ. Sci. 2023, 16, 4373.

[19] S. Shaik , J. Kundu , Y. Yuan , W. Chung , D. Han , U. Lee , H. Huang , S.‐I. Choi , Adv. Energy Mater. 2024, 14, 2401956.

[20] Y. Leng , G. Chen , A. J. Mendoza , T. B. Tighe , M. A. Hickner , C.‐Y. Wang , J. Am. Chem. Soc. 2012, 134, 9054. 22587676 10.1021/ja302439z

[21] D. Li , E. J. Park , W. Zhu , Q. Shi , Y. Zhou , H. Tian , Y. Lin , A. Serov , B. Zulevi , E. D. Baca , C. Fujimoto , H. T. Chung , Y. S. Kim , Nat. Energy 2020, 5, 378.

[22] W. Song , K. Peng , W. Xu , X. Liu , H. Zhang , X. Liang , B. Ye , H. Zhang , Z. Yang , L. Wu , X. Ge , T. Xu , Nat. Commun. 2023, 14, 2732. 37169752 10.1038/s41467-023-38350-7 PMC10175247

[23] R. Soni , S. Miyanishi , H. Kuroki , T. Yamaguchi , ACS Appl. Energy Mater. 2021, 4, 1053.

[24] J. Xiao , A. M. Oliveira , L. Wang , Y. Zhao , T. Wang , J. Wang , B. P. Setzler , Y. Yan , ACS Catal. 2021, 11, 264.

[25] M. S. Lemcke , S. Loos , N. Menzel , M. Bron , ChemElectroChem 2024, 11, 21.

[26] D. Chanda , J. Hnát , T. Bystron , M. Paidar , K. Bouzek , J. Power Sources 2017, 347, 247.

[27] J. Hyun , S. Hwan Yang , D. Wook Lee , E. Oh , H. Bae , M. Suc Cha , G. Doo , J. Yong Lee , H.‐T. Kim , Chem. Eng. J. 2023, 469, 143919.

[28] S. Campagna Zignani , M. Lo Faro , A. Carbone , C. Italiano , S. Trocino , G. Monforte , A. S. Aricò , Electrochim. Acta 2022, 413, 140078.

[29] G. A. Lindquist , Q. Xu , S. Z. Oener , S. W. Boettcher , Joule 2020, 4, 2549.

[30] W. Chen , H. Wang , Y. Li , Y. Liu , J. Sun , S. Lee , J.‐S. Lee , Y. Cui , ACS Cent. Sci. 2015, 1, 244. 27162978 10.1021/acscentsci.5b00227 PMC4827502

[31] I. Galkina , A. Y. Faid , W. Jiang , F. Scheepers , P. Borowski , S. Sunde , M. Shviro , W. Lehnert , A. K. Mechler , Small 2024, 20, 2311047. 10.1002/smll.202311047 38269475

[32] R. A. Krivina , G. A. Lindquist , M. C. Yang , A. K. Cook , C. H. Hendon , A. R. Motz , C. Capuano , K. E. Ayers , J. E. Hutchison , S. W. Boettcher , ACS Appl. Mater. Interfaces 2022, 14, 18261. 35435656 10.1021/acsami.1c22472

[33] R. A. Krivina , G. A. Lindquist , S. R. Beaudoin , T. N. Stovall , W. L. Thompson , L. P. Twight , D. Marsh , J. Grzyb , K. Fabrizio , J. E. Hutchison , S. W. Boettcher , Adv Mater 2022, 34, e2203033. 35790033 10.1002/adma.202203033

[34] B. Mayerhöfer , K. Ehelebe , F. D. Speck , M. Bierling , J. Bender , J. A. Kerres , K. J. J. Mayrhofer , S. Cherevko , R. Peach , S. Thiele , J. Mater. Chem. A 2021, 9, 14285.

[35] E. Cossar , F. Murphy , J. Walia , A. Weck , E. A. Baranova , ACS Appl. Energy Mater. 2022, 5, 9938.

[36] E. K. Volk , A. L. Clauser , M. E. Kreider , D. D. Soetrisno , S. Khandavalli , J. D. Sugar , S. Kwon , S. M. Alia , ACS Electrochem. 2024, 1, 239. 39935602 10.1021/acselectrochem.4c00061 PMC11808641

[37] E. K. Volk , M. E. Kreider , S. Kwon , S. M. Alia , EES Catal. 2024, 2, 109.

[38] N. Du , C. Roy , R. Peach , M. Turnbull , S. Thiele , C. Bock , Chem. Rev. 2022, 122, 11830. 35442645 10.1021/acs.chemrev.1c00854 PMC9284563

[39] Y. Jia , Y. Li , Q. Zhang , S. Yasin , X. Zheng , K. Ma , Z. Hua , J. Shi , C. Gu , Y. Dou , S. Dou , Carbon Energy 2024, 6, 528.

[40] B. Mayerhöfer , F. D. Speck , M. Hegelheimer , M. Bierling , D. Abbas , D. McLaughlin , S. Cherevko , S. Thiele , R. Peach , Int. J. Hydrogen Energy 2022, 47, 4304.

[41] J. Lei , Z. Wang , Y. Zhang , M. Ju , H. Fei , S. Wang , C. Fu , X. Yuan , Q. Fu , M. U. Farid , H. Kong , A. K. An , R. Deng , F. Liu , J. Wang , Carbon Neutrality 2024, 3, 1.

[42] T. B. Ferriday , P. H. Middleton , M. L. Kolhe , Energies 2021, 14, 8535.

[43] L. J. Titheridge , S. Sharma , A. Soisson , C. Tiffin , C. Roth , A. T. Marshall , ACS Electrochem. 2025, 1, 951.

[44] E. Cossar , A. O. Barnett , F. Seland , R. Safari , G. A. Botton , E. A. Baranova , J. Power Sources 2021, 514, 230563.

[45] G. Huang , M. Mandal , N. U. Hassan , K. Groenhout , A. Dobbs , W. E. Mustain , P. A. Kohl , J. Electrochem. Soc. 2020, 167, 164514.

[46] G. Huang , M. Mandal , N. U. Hassan , K. Groenhout , A. Dobbs , W. E. Mustain , P. A. Kohl , J. Electrochem. Soc. 2021, 168, 024503.

[47] S. Koch , P. A. Heizmann , S. K. Kilian , B. Britton , S. Holdcroft , M. Breitwieser , S. Vierrath , J. Mater. Chem. A 2021, 9, 15744.

[48] Y. S. Park , M. J. Jang , J.‐Y. Jeong , J. Lee , J. Jeong , C. Kim , J. Yang , S. M. Choi , Int. J. Energy Res. 2023, 2023, 1.

[49] X. Zhang , Y. Li , W. Zhao , J. Guo , P. Yin , T. Ling , Int. J. Miner. Metall. Mater. 2023, 30, 2259.

[50] M. E. Kreider , H. Yu , L. Osmieri , M. R. Parimuha , K. S. Reeves , D. H. Marin , R. T. Hannagan , E. K. Volk , T. F. Jaramillo , J. L. Young , P. Zelenay , S. M. Alia , ACS Catal. 2024, 14, 10806. 39050897 10.1021/acscatal.4c02932 PMC11264204

[51] L. Wang , T. Weissbach , R. Reissner , A. Ansar , A. S. Gago , S. Holdcroft , K. A. Friedrich , ACS Appl. Energy Mater. 2019, 2, 7903.

[52] F. Razmjooei , T. Morawietz , E. Taghizadeh , E. Hadjixenophontos , L. Mues , M. Gerle , B. D. Wood , C. Harms , A. S. Gago , S. A. Ansar , K. A. Friedrich , Joule 2021, 5, 1776.

[53] V. Wilke , M. Rivera , T. Morawietz , N. Sata , L. Mues , M. Hegelheimer , A. Maljusch , P. Borowski , G. Schmid , C. Y. Thum , M. Klingenhof , P. Strasser , A. Karl , S. Basak , J.‐P. Poc , R.‐A. Eichel , A. S. Gago , K. A. Friedrich , Electrochem. Sci. Adv. 2024, 5, 202400036.

[54] M. Plevová , J. Hnát , J. Žitka , L. Pavlovec , M. Otmar , K. Bouzek , J. Power Sources 2022, 539, 231476.

[55] S. Koch , L. Metzler , S. K. Kilian , P. A. Heizmann , F. Lombeck , M. Breitwieser , S. Vierrath , Adv. Sustainable Syst. 2023, 7, 2200332.

[56] S. Ding , Z. Li , G. Lin , L. Wang , A. Dong , L. Sun , ACS Energy Lett. 2024, 9, 3719.

[57] L. Wan , J. Liu , Z. Xu , Q. Xu , M. Pang , P. Wang , B. Wang , Small 2022, 18, e2200380. 35491509 10.1002/smll.202200380

[58] S. Appelhaus , L. Ritz , S.‐V. Pape , F. Lohmann‐Richters , M. R. Kraglund , J. O. Jensen , F. Massari , M. Boroomandnia , M. Romanò , J. Albers , C. Kubeil , C. Bernäcker , M. S. Lemcke , N. Menzel , G. Bender , B. Chen , S. Holdcroft , R. Delmelle , J. Proost , J. Hnát , P. Kauranen , V. Ruuskanen , T. Viinanen , M. Müller , T. Turek , M. Shviro , Int. J. Hydrogen Energy 2024, 95, 1004.

[59] G. Bender , M. Carmo , T. Smolinka , A. Gago , N. Danilovic , M. Mueller , F. Ganci , A. Fallisch , P. Lettenmeier , K. A. Friedrich , K. Ayers , B. Pivovar , J. Mergel , D. Stolten , Int. J. Hydrogen Energy 2019, 44, 9174.

[60] Z. Liu , S. D. Sajjad , Y. Gao , H. Yang , J. J. Kaczur , R. I. Masel , Int. J. Hydrogen Energy 2017, 42, 29661.

[61] P.‐S. Jhu , C.‐W. Chang , C.‐C. Cheng , Y.‐C. Ting , T.‐Y. Lin , F.‐Y. Yen , P.‐W. Chen , S.‐Y. Lu , Nano Energy 2024, 126, 109703.

[62] P. Paknahad , M. Askari , M. Ghorbanzadeh , Appl. Phys. A 2015, 119, 727.

