# Supplementary material for: Elucidating the Degradation Behavior of a 25 cm2 Pure‐Water‐Fed Non‐Precious Metal Anion Exchange Membrane Water Electrolyzer Cell
Source: Small. 2025 Dec 3;22(2):e06262. doi: 10.1002/smll.202506262 (PMC12781622; doi:10.1002/smll.202506262)
Supplement: Supplementary file 1 — Supporting Information [file SMLL-22-e06262-s001.pdf]

NANO · MICRO small

Supporting Information

for *Small*, DOI 10.1002/smll.202506262

Elucidating the Degradation Behavior of a 25 cm^2 Pure-Water-Fed Non-Precious Metal Anion Exchange Membrane Water Electrolyzer Cell

Michelle S. Lemcke, Robert Göckeritz, Alexander Müller, Kerstin Witte-Bodnar, Wolfram Münchgesang and Michael Bron*

Supporting Information

Elucidating the Degradation Behavior of a $25\,\mathrm{cm}^2$ Pure-Water-Fed Non-Precious Metal Anion Exchange Membrane Electrolyzer Cell

Michelle Sophie Lemcke,*[a,b] Robert Göckeritz,[c] Alexander Müller,[c] Kerstin Witte-Bodnar,[c,d] Wolfram Münchgesang,[a] Michael Bron[b]

[a] Fraunhofer Institute for Wind Energy Systems IWES, Am Seedeich 45, 27572 Bremerhaven, Germany
*E-mail: michelle.sophie.lemcke@iwes.fraunhofer.de

[b] Martin Luther University Halle-Wittenberg, Institute of Chemistry, Von-Danckelmann-Platz 4, 06120 Halle (Saale), Germany

[c] Fraunhofer Institute for Microstructure of Materials and Systems IMWS, Walter-Huelse-Strasse 1, 06120 Halle (Saale), Germany

[d] Hochschule Anhalt, University of Applied Science, Bernburger Strasse 55, 06366 Köthen, Germany

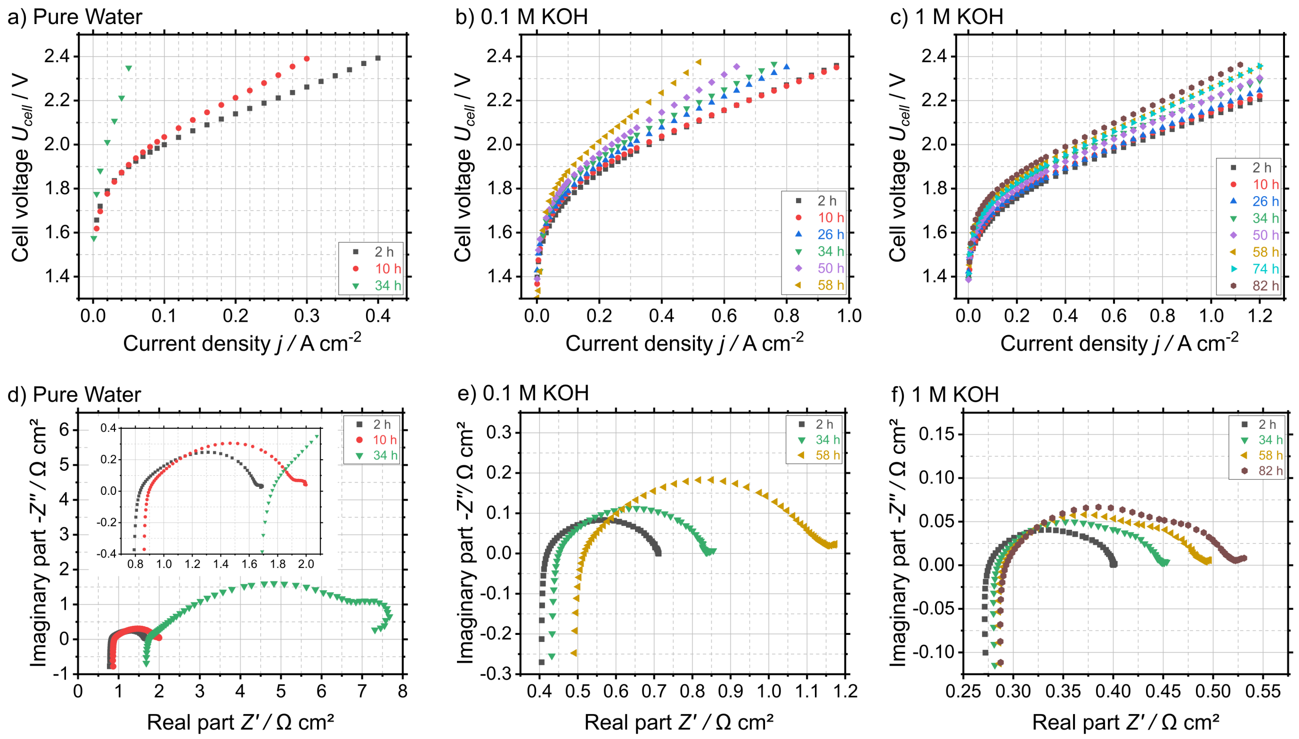

Figure S1. Electrochemical evaluation of the developed non-precious metal AEMWE cell with an electrode area of 25 cm^2 operated at 60 °C. Polarization curves of the AEMWE cell operated in a) pure water, b) 0.1 M KOH and c) 1.0 M KOH. Nyquist plots obtained from EIS at 2 V from 100 kHz to 100 mHz in d) pure water, e) 0.1 M KOH and f) 1.0 M KOH.

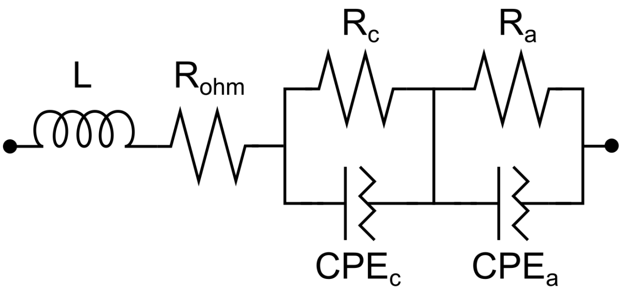

Figure S2. Equivalent electric circuit model consisting of a series connection of an inductor (L), an ohmic resistance (R_{ohm}) and two parallel branches, each comprising a resistor and a constant phase element (CPE).

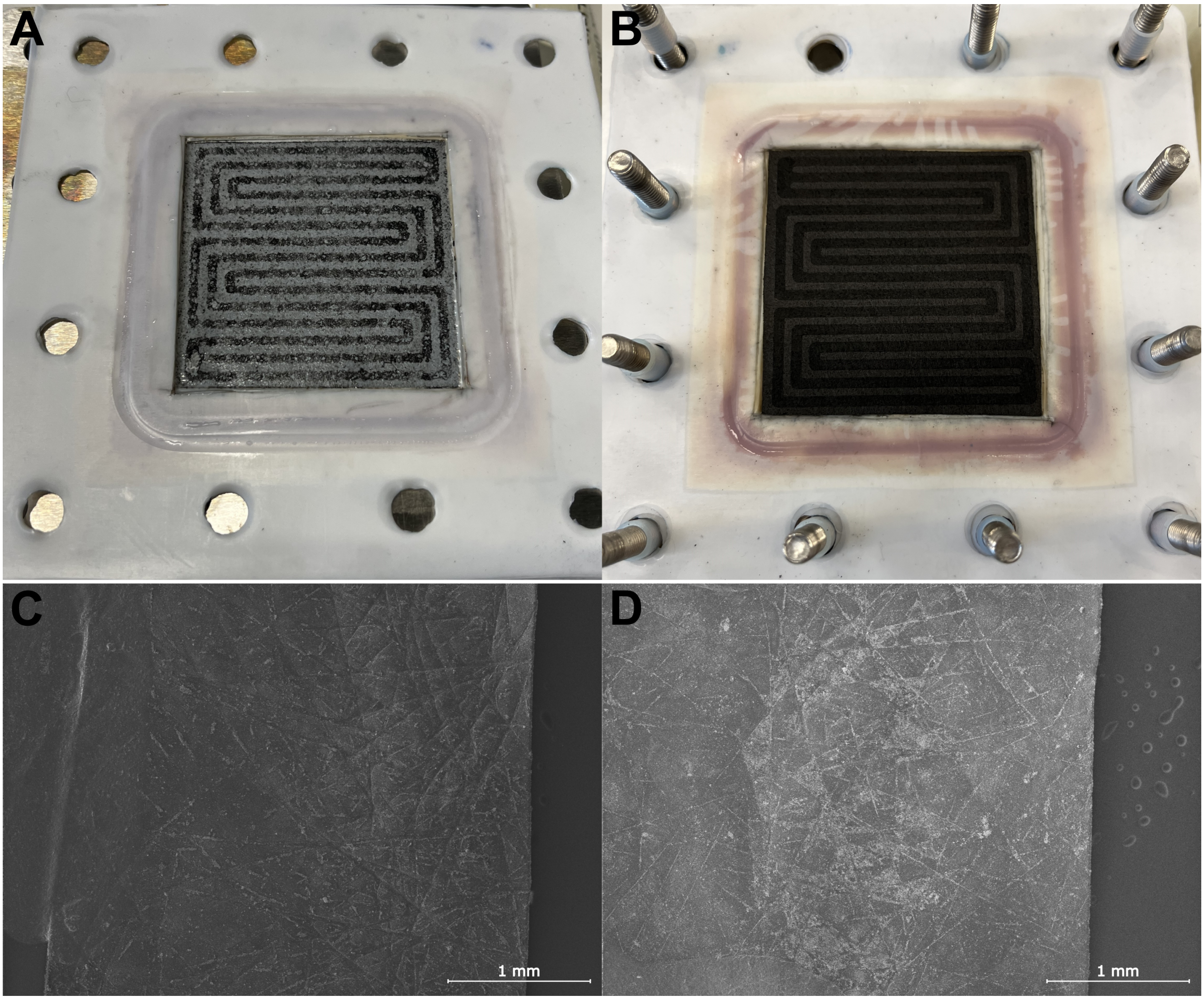

Figure S3. Photographs of the AEMWE cell after operation showing A) membrane with catalyst leftovers of the anode in the shape of the flow field and B) cathode electrode placed on the membrane stamped by the flow field. Top-view SEM images of the post mortem membrane stamped by the flow fields and fibrous PTLs due to the contact pressure applied in the AEMWE cell showing C) interface to the cathode and D) interface to the anode.

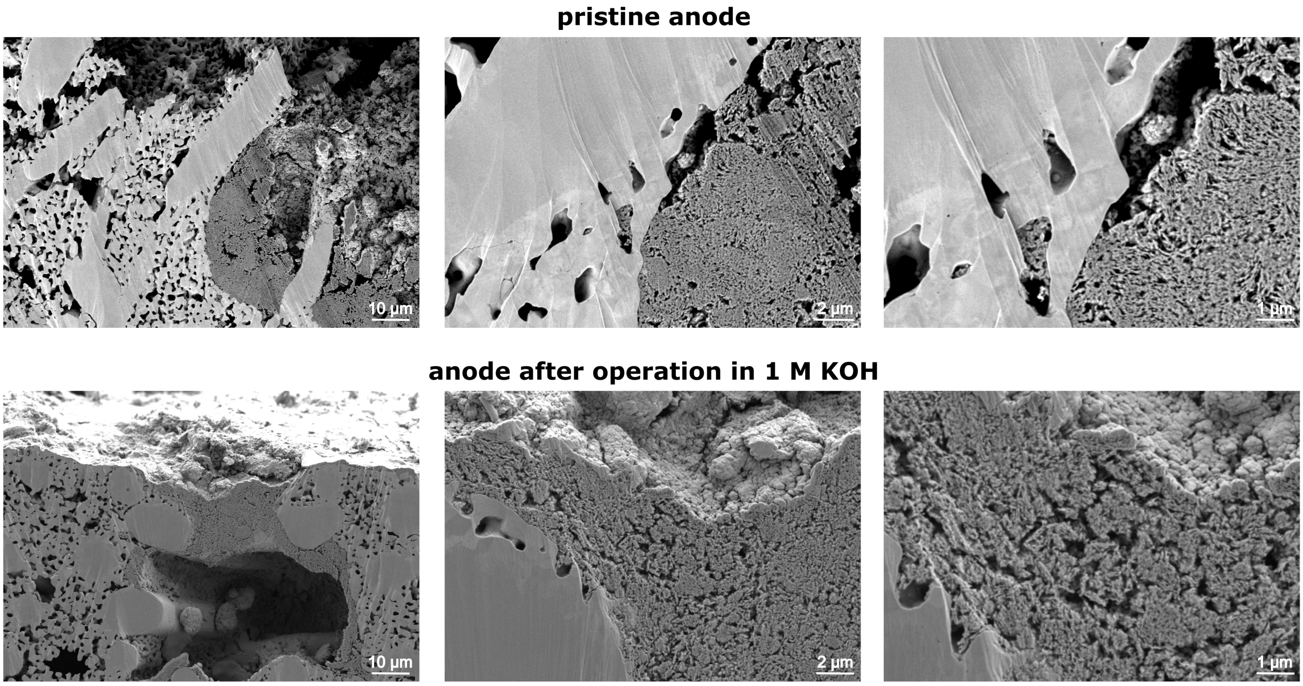

Figure S4. Cross-sectional SEM images with different magnifications of the anode catalyst layer at the pristine state (top row) and at the post mortem state after operation in 1.0 M KOH (bottom row).

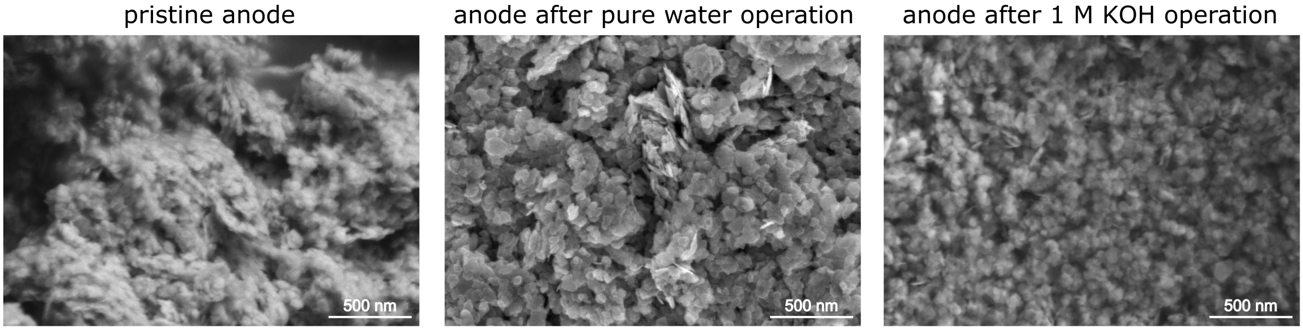

Figure S5. Higher-resolution top-view SEM images of the $Cu_{0.6}Mn_{0.3}Co_{2.1}O_4$ anode at the pristine state (left) as well as at the post mortem state after operation in pure water (middle) and in 1.0 M KOH (right).

Table S1. EDS analysis results of the cross section of the pristine anode and the post mortem anode operated in 1.0 M KOH: A) elements of anode electrode, B) elements of OER catalyst (excluding oxygen) and C) elements of catalyst layer. Cu, Co, Mn and O are markers for the $Cu_{0.6}Mn_{0.3}Co_{2.1}O_4$ catalyst. C is a marker for the ionomer in the catalyst layer. Ni, Fe and Cr are markers for the Ni-coated 316L-PTL.
Comparing the pristine and post mortem states, Tables S1A-C show a relative reduction of Cu content in the catalyst over time, indicating Cu dissolution. Tables S1A and S1C show a relative reduction of C content in the catalyst layer from pristine to post mortem state, indicating ionomer loss.

**A**			**B**			**C**		
Element	pristine / wt.-%	post mortem / wt.-%	Element	pristine / wt.-%	post mortem / wt.-%	Element	pristine / wt.-%	post mortem / wt.-%
Co	5.2	6.0	Co	69.7	75.2	Co	33.7	38.9
Cu	1.4	1.0	Cu	19.0	12.5	Cu	9.2	6.4
Mn	0.8	1.0	Mn	11.2	12.3	Mn	5.4	6.4
O	3.3	3.6				O	21.2	23.4
C	4.7	3.9				C	30.4	24.9
Ni	50.9	43.9						
Fe	25.7	30.9						
Cr	6.7	8.0						

Table S2. EDS analysis results of the top view (catalyst layer) of the pristine anode, the post mortem anode operated in pure water and the post mortem anode operated in 1.0 M KOH: A) elements of OER catalyst (excluding oxygen) and B) elements of catalyst layer. Cu, Co, Mn and O are markers for the $Cu_{0.6}Mn_{0.3}Co_{2.1}O_4$ catalyst. C is a marker for the ionomer in the catalyst layer. Ni, Fe and Cr are markers for the Ni-coated 316L-PTL.
Comparing the pristine and post mortem states, Table S2A shows a relative reduction of Cu content in the catalyst over time for both post mortem anodes, indicating Cu dissolution. Table S2B shows a relative reduction of C content in the catalyst layer over time for both post mortem anodes, indicating ionomer loss.

**A**				**B**			
Element	pristine / wt.-%	post mortem pure water / wt.-%	post mortem 1.0 M KOH / wt.-%	Element	pristine / wt.-%	post mortem pure water / wt.-%	post mortem 1.0 M KOH / wt.-%
Co	70.3	73.1	75.7	Co	28.6	46.8	49.2
Cu	21.0	17.9	14.8	Cu	8.5	11.5	9.7
Mn	8.8	9.0	9.5	Mn	3.6	5.8	6.2
				O	29.1	28.6	27.6
				C	30.2	7.3	7.4

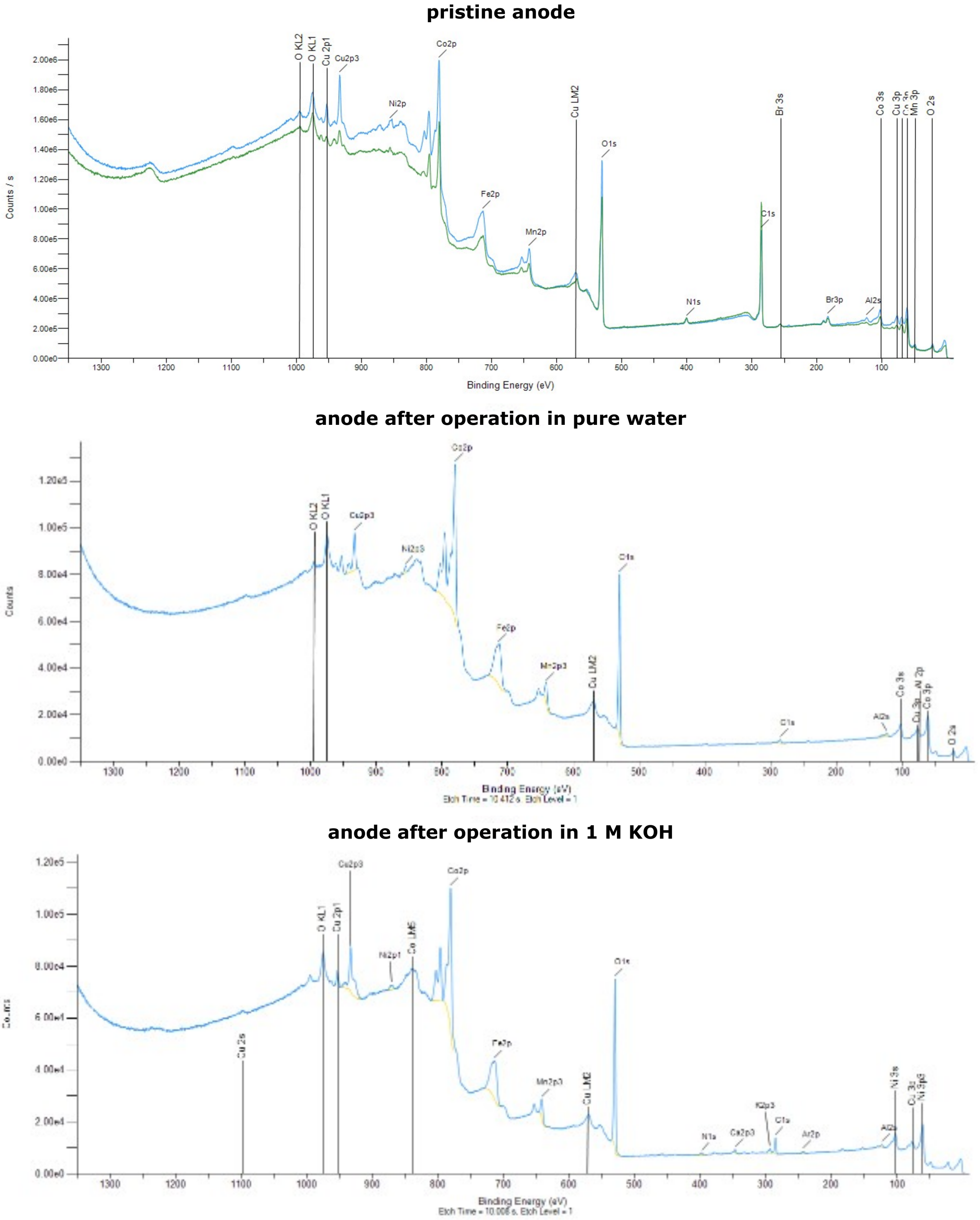

Figure S6. XPS survey spectra of the pristine anode, the post mortem anode operated in pure water and the post mortem anode operated in 1.0 M KOH. Cu, Co, Mn and O are markers for the $Cu_{0.6}Mn_{0.3}Co_{2.1}O_4$ catalyst. C and N can be markers for the ionomer in the catalyst layer. Ni, Fe and Cr are markers for the Ni-coated 316L-PTL. The corresponding molar fractions can be found in the following table, Table S3.

Table S3. XPS analysis results of A) the pristine anode, B) the post mortem anode operated in pure water and C) the post mortem anode operated in 1.0 M KOH, at two different etch times (0 s and 10 s). Cu, Co, Mn and O are markers for the $Cu_{0.6}Mn_{0.3}Co_{2.1}O_4$ catalyst. C and N can be markers for the ionomer in the catalyst layer. Ni, Fe and Cr are markers for the Ni-coated 316L-PTL.

Comparing the pristine and post mortem states, Table S3 shows a relative reduction of C content in the catalyst layer over time for both post mortem anodes. It also shows a relative reduction of N content in the catalyst layer for the post mortem anode operated in 1.0 M KOH and no detectable N content in the catalyst layer of the post mortem anode operated in pure water, indicating ionomer loss. These results are in alignment with the findings from the electrochemical and SEM-EDS analyses. It should be noted that only the uppermost atomic layers of the sample are examined in XPS analysis and it does not necessarily represent the entire catalyst layer. To reach more definitive and universal conclusions, high-resolution spectra of each element of the catalyst layer as well as XPS analysis from multiple regions are needed.

**A**			**B**			**C**		
pristine anode element	0 s / at.-%	10 s / at.-%	pure water element	0 s / at.-%	10 s / at.-%	1.0 M KOH element	0 s / at.-%	10 s / at.-%
C1s	38.71	51.2	C1s	12.9	4.1	C1s	14.6	9.2
Co2p	7.0	5.7	Co2p	18.5	27.0	Co2p	14.1	22.1
Fe2p	8.2	3.6	Fe2p	9.1	10.5	Fe2p	7.8	9.0
O1s	31.0	30.0	O1s	47.0	43.6	O1s	49.0	42.9
Al2s	3.7	2.3	Al2s	5.3	6.7	Al2s	1.0	2.2
Br3p	1.1	1.2	Br3p	-	-	Br3p	-	-
Cu2p3	2.1	0.7	Cu2p3	3.3	4.2	Cu2p3	5.6	6.8
Mn2p	4.9	2.7	Mn2p3	2.9	3.3	Mn2p3	2.5	2.8
**N1s**	**2.1**	**2.0**	**N1s**	**-**	**-**	**N1s**	**1.5**	**1.4**
Ni2p	1.2	0.7	Ni2p3	1.0	0.7	Ni2p1	1.5	1.5
Ar2p	-	-	Ar2p	-	-	Ar2p	0.2	0.6
Ca2p3	-	-	Ca2p3	-	-	Ca2p3	0.5	0.4
K2p3	-	-	K2p3	-	-	K2p3	1.8	1.1

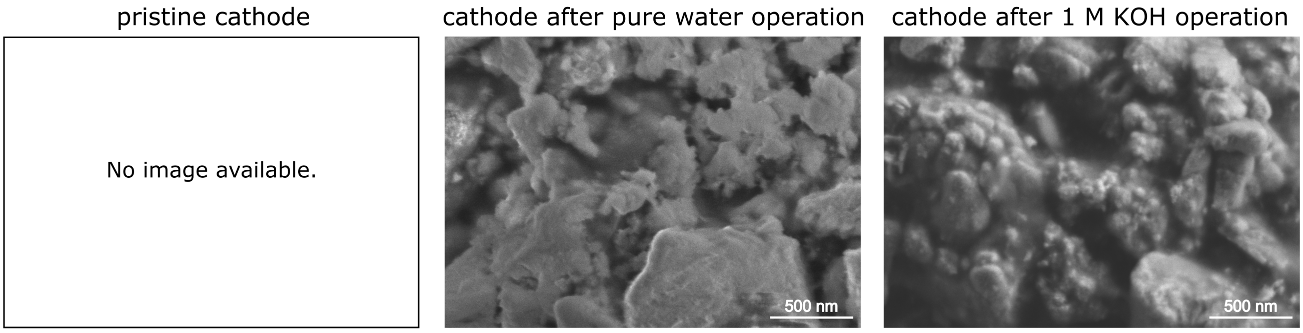

Figure S7. Higher-resolution top-view SEM images of the Raney-Nickel cathode at the post mortem state after operation in pure water (middle) and in 1.0 M KOH (right). No image available for the pristine state (left).

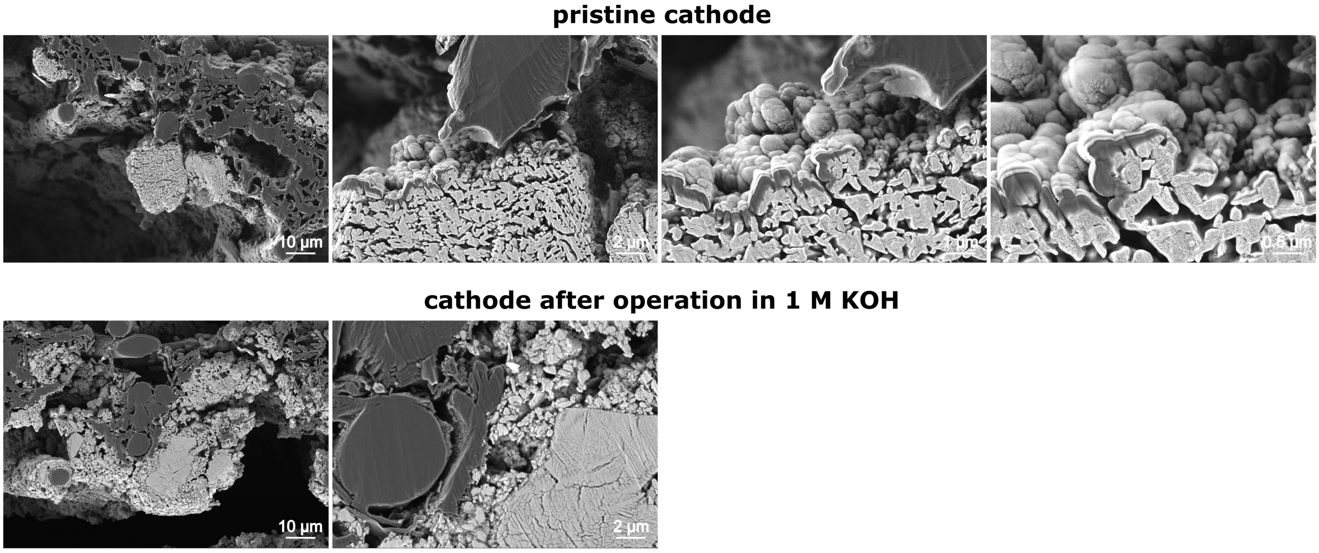

Figure S8. Cross-sectional SEM images with different magnifications of the cathode catalyst layer at the pristine state (top row) and at the post mortem state after operation in 1.0 M KOH (bottom row).

Table S4. EDS analysis results of the cross section of the pristine cathode and the post mortem cathode operated in 1.0 M KOH: A) elements of cathode electrode, B) elements of HER catalyst and C) elements of HER catalyst and oxygen. Ni and Fe are markers for the Raney-Nickel catalyst. C is the marker for the carbon PTL. (C is also marker for the ionomer in the catalyst layer. However, the proportion of the PTL in the C signal is significantly greater than that of the ionomer.)

As shown in Table S4A, Cr and Cu were detected in the post mortem cathode but not in the pristine cathode, supporting the assumption of Cu dissolution from the anode electrode and Cr dissolution from the anode PTL. It is noted that the percentage of C is lower in the post mortem cathode due to the higher catalyst coverage of the PTL compared to the pristine cathode. Comparing the pristine and post mortem states of the catalyst, Table S4B shows that the ratio of Ni and Fe stays nearly the same. However, the ratio between catalyst (Ni, Fe) and oxygen changes over time, as shown in Table S4C. This relative increment of O content in the catalyst layer from pristine to post mortem state supports the argumentation that hydride formation occurred in the cathode during operation.

**A**			**B**			**C**		
Element	pristine / wt.-%	post mortem / wt.-%	Element	pristine / wt.-%	post mortem / wt.-%	Element	pristine / wt.-%	post mortem / wt.-%
Ni	21.0	30.9	Ni	89.1	88.6	Ni	70.6	66.6
Fe	2.6	4.0	Fe	10.9	11.4	Fe	8.7	8.6
O	6.2	11.5				O	20.7	24.8
C	66.4	49.7						
Cr	-	1.5						
Cu	-	0.3						

Table S5. EDS analysis results of the top view (catalyst layer) of the pristine cathode and the post mortem cathode operated in pure water: A) elements of cathode electrode, B) elements of HER catalyst and C) elements of HER catalyst and oxygen. Ni and Fe are markers for the Raney-Nickel catalyst. C is the marker for the carbon PTL. (C is also marker for the ionomer in the catalyst layer. However, the proportion of the PTL in the C signal is significantly greater than that of the ionomer.)
As shown in Table S5A, Cr and Cu were detected in the pure-water-fed post mortem cathode but not in the pristine cathode, supporting the assumption of Cu dissolution from the anode electrode and Cr dissolution from the anode PTL. Comparing the pristine and post mortem states of the catalyst, Table S5B shows that the ratio of Ni and Fe stays the same. However, the ratio between catalyst (Ni, Fe) and oxygen changes over time, as shown in Table S5C. This relative increment of O content in the catalyst layer from pristine to post mortem state supports the argumentation that hydride formation occurred in the cathode during operation.

**A**			**B**			**C**		
Element	pristine / wt.-%	post mortem / wt.-%	Element	pristine / wt.-%	post mortem / wt.-%	Element	pristine / wt.-%	post mortem / wt.-%
Ni	17.8	16.4	Ni	89.7	89.4	Ni	62.1	51.5
Fe	2.1	2.0	Fe	10.3	10.6	Fe	7.2	6.1
O	8.8	13.5				O	30.8	42.4
C	68.0	66.8						
Cr	-	0.2						
Cu	-	0.3						

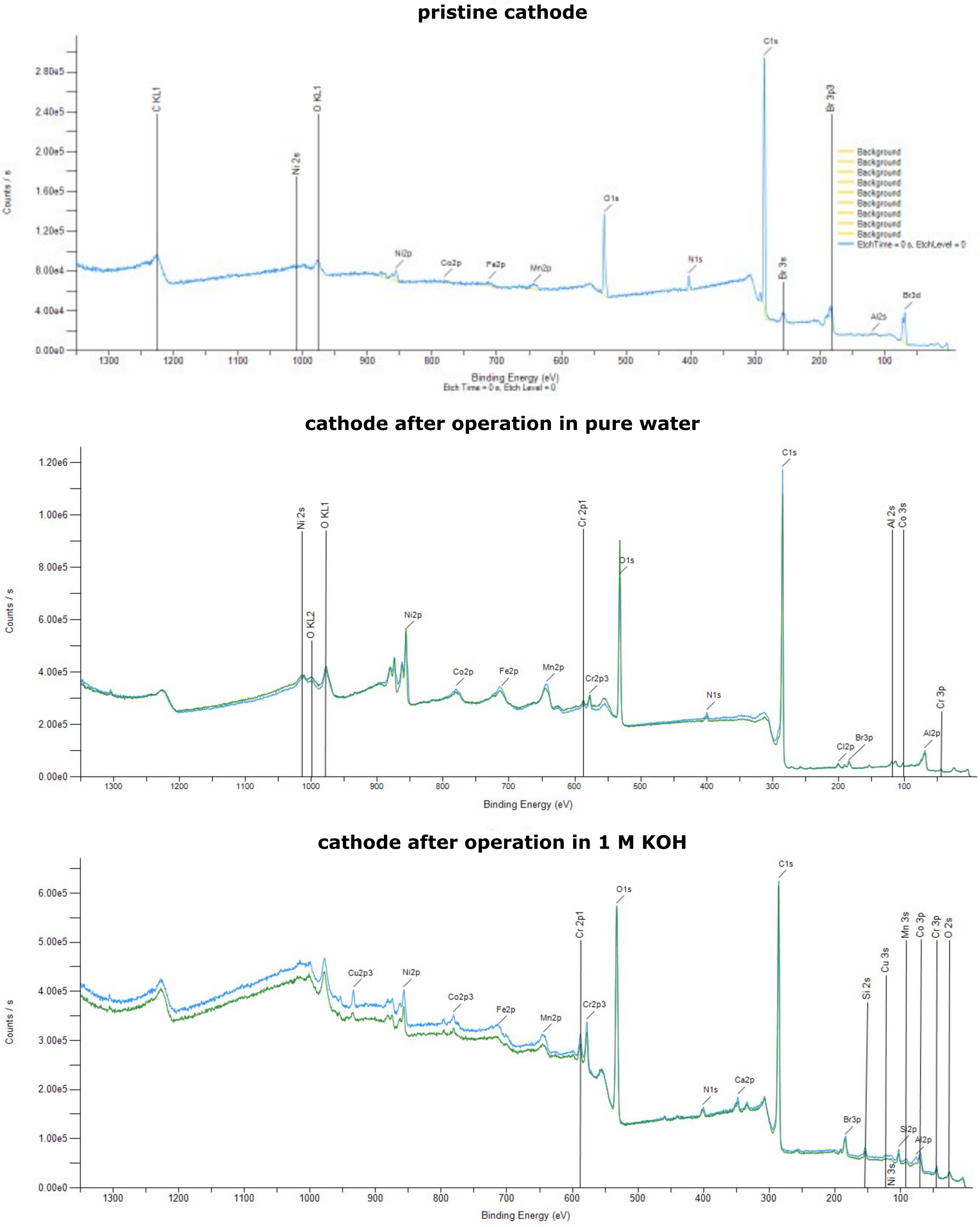

Figure S9. XPS survey spectra of the pristine cathode, the post mortem cathode operated in pure water and the post mortem cathode operated in 1.0 M KOH. Ni and Fe are markers for the Raney-Nickel catalyst. C and N can be markers for the ionomer in the catalyst layer. C is also the marker for the carbon PTL. The corresponding molar fractions can be found in the following table, Table S6.

Table S6. XPS analysis results of A) the pristine cathode, B) the post mortem cathode operated in pure water and C) the post mortem cathode operated in 1.0 M KOH, at two different etch times (0 s and 10 s). Ni and Fe are markers for the Raney-Nickel catalyst. C and N can be markers for the ionomer in the catalyst layer. C is also the marker for the carbon PTL.
Comparing the pristine and post mortem states, Table S6 shows a relative reduction of N content in the catalyst layer for both post mortem cathodes, indicating ionomer loss. It should be noted that only the uppermost atomic layers of the sample are examined in XPS analysis and it does not necessarily represent the entire catalyst layer. This is in alignment with the findings from the electrochemical and SEM-EDS analyses. To reach more definitive and universal conclusions, high-resolution spectra of each element of the catalyst layer as well as XPS analysis from multiple regions are needed.

**A**			**B**			**C**		
pristine cathode element	0 s / at.-%	10 s / at.-%	pure water element	0 s / at.-%	10 s / at.-%	1.0 M KOH element	0 s / at.-%	10 s / at.-%
C1s	78.0	79.0	C1s	59.8	57.5	C1s	60.7	62.8
O1s	11.1	7.4	O1s	15.2	19.3	O1s	23.8	25.4
Al2s	0.3	0.6	Al2p	14.5	13.2	Al2p	1.9	0.6
Br3d	5.2	5.0	Br3p	0.4	0.4	Br3p	1.0	1.0
**N1s**	**3.3**	**3.4**	**N1s**	**1.2**	**1.0**	**N1s**	**1.6**	**1.7**
Ni2p	1.0	2.2	Ni2p	3.7	3.9	Ni2p	1.7	1.4
Co2p	0.2	0.5	Co2p	1.2	1.0	Co2p3	1.1	0.7
Fe2p	0.4	0.7	Fe2p	1.2	1.4	Fe2p	1.0	0.6
Mn2p	0.6	1.4	Mn2p	1.9	1.5	Mn2p	1.3	0.8
Ca2p	-	-	Ca2p	-	-	Ca2p	0.8	0.7
Cr2p3	-	-	Cr2p3	0.5	0.5	Cr2p3	2.3	1.9
Cu2p3	-	-	Cu2p3	-	-	Cu2p3	0.4	0.2
Si2p	-	-	Si2p	-	-	Si2p	2.5	2.1
Cl2p	-	-	Cl2p	0.4	0.4	Cl2p	-	-

Table S7. ICP-MS results of the 1.0 M KOH electrolyte before and after the single cell operation. The mean value with standard deviation derived from three measurements is given.

electrolyte	Cu / $\mu g\,L^{-1}$	Cr / $\mu g\,L^{-1}$
pristine	n.a.	n.a.
after operation (anode side)	307 ± 10	656 ± 20
after operation (cathode side)	4 ± 1	17.7 ± 0.3

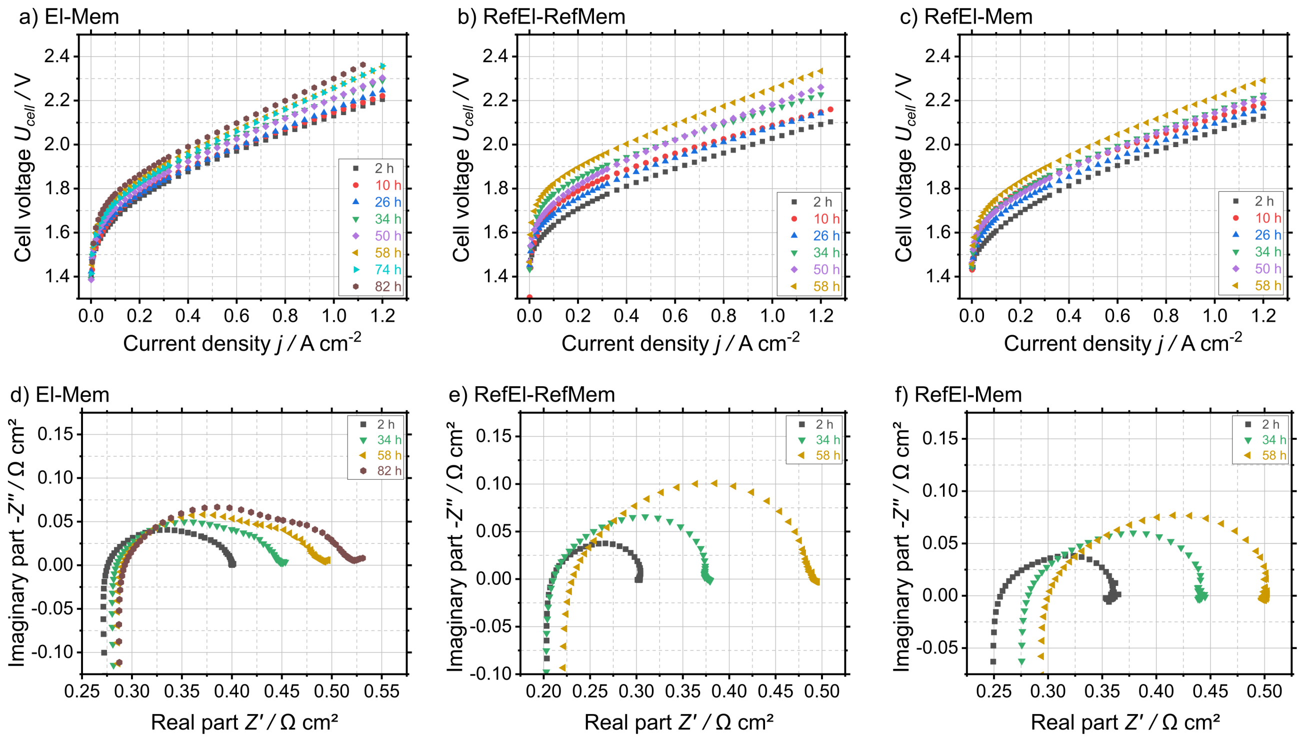

Figure S10. Electrochemical evaluation of AEMWE cells with developed and commercially available electrodes and membranes with electrode areas of 25 cm^2 operated in 1.0 M KOH at 60 °C. Polarization curves of AEMWE cells a) El-Mem, b) RefEl-RefMem and c) RefEl-Mem. Nyquist plots obtained from EIS at 2 V from 100 kHz to 100 mHz for AEMWE cells d) El-Mem, e) RefEl-RefMem and f) RefEl-Mem.

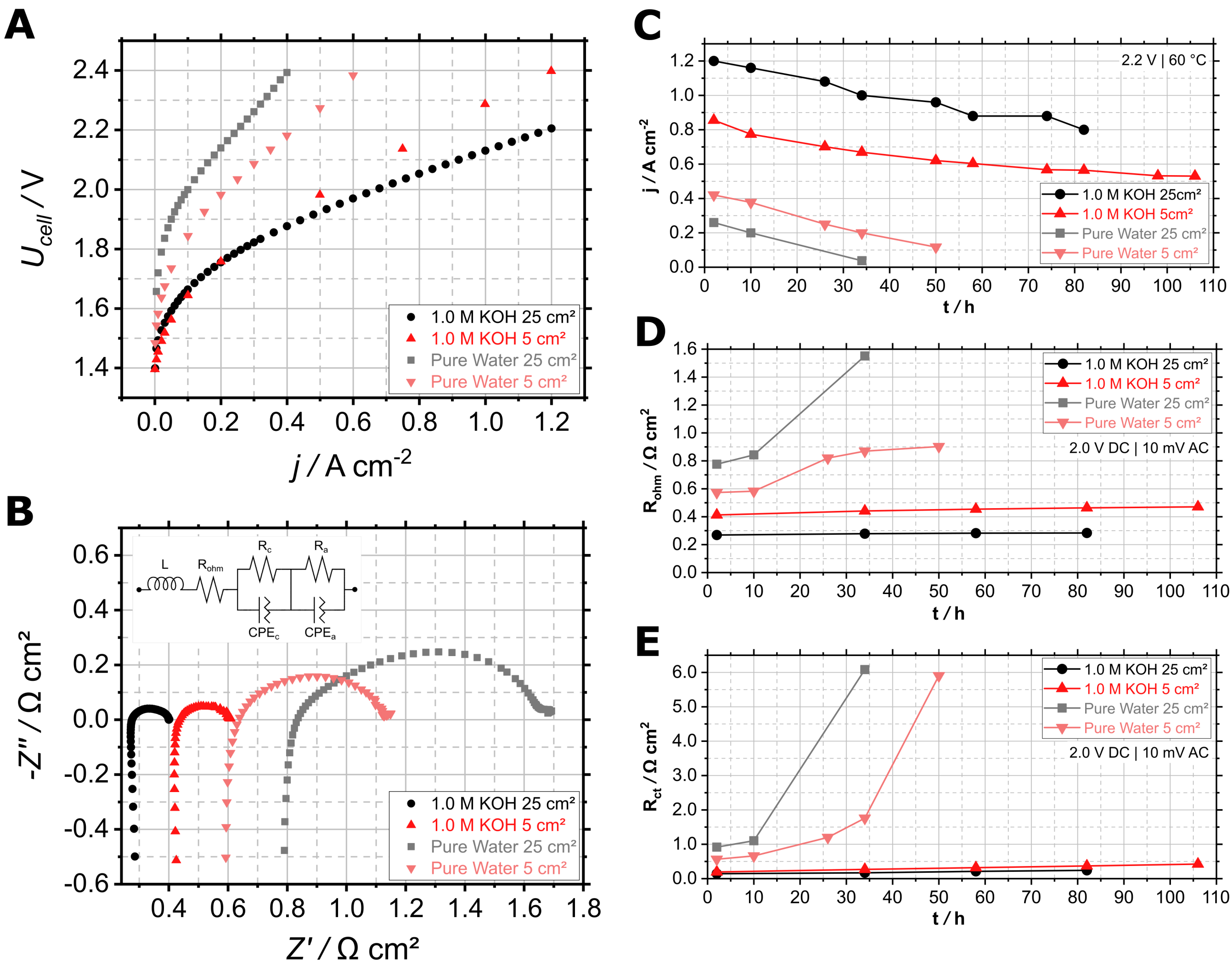

Figure S11. Comparison of non-precious metal AEMWE cells with electrode areas of 5 cm^2 and 25 cm^2, operated in pure water and 1.0 M KOH: A) Polarization curves; B) Nyquist plots obtained from EIS at 2 V from 100 kHz to 100 mHz; C) time-resolved change in current density j at 2.2 V; time-dependent change in D) ohmic resistance R_{ohm} and E) charge transfer resistance R_{ct}, both obtained from EIS at 2.0 V.
